# Unraveling Cadmium Toxicity in *Trifolium repens* L. Seedling: Insight into Regulatory Mechanisms Using Comparative Transcriptomics Combined with Physiological Analyses

**DOI:** 10.3390/ijms23094612

**Published:** 2022-04-21

**Authors:** Feifei Wu, Jinwan Fan, Xiuwen Ye, Lili Yang, Ruchang Hu, Jieyu Ma, Sainan Ma, Dandan Li, Jiqiong Zhou, Gang Nie, Xinquan Zhang

**Affiliations:** Department of Forage Science, College of Grassland Science and Technology, Sichuan Agricultural University, Chengdu 611130, China; wufeifei_wu@163.com (F.W.); 15513999795@163.com (J.F.); xiuwen_ye@163.com (X.Y.); yanlijms@foxmail.com (L.Y.); huuruchang@163.com (R.H.); majieyu0224@163.com (J.M.); masainan1997@163.com (S.M.); lidandan@sicau.edu.cn (D.L.); jiqiong_zhou@outlook.com (J.Z.)

**Keywords:** *Trifolium repens*, Cadmium, transcriptomic, physiological, cell wall, GSH metabolism

## Abstract

*Trifolium repens* (*T. repens*) can accumulate significant amounts of heavy metal ions, and has strong adaptability to wide environmental conditions, and relatively large biomass, which is considered a potential plant for phytoremediation. However, the molecular mechanisms of *T. repens* involved in Cd tolerance have not yet been studied in detail. This study was conducted to examine the integrative responses of *T. repens* exposed to a high-level CdCl_2_ by investigating the physiological and transcriptomic analyses. The results suggested that *T. repens* seedlings had a high degree of tolerance to Cd treatment. The roots accumulated higher Cd concentration than leaves and were mainly distributed in the cell wall. The content of MDA, soluble protein, the relative electrolyte leakage, and three antioxidant enzymes (POD, SOD, and APX) was increased with the Cd treatment time increasing, but the CAT enzymes contents were decreased in roots. Furthermore, the transcriptome analysis demonstrated that the differentially expressed genes (DEGs) mainly enriched in the glutathione (GSH) metabolism pathway and the phenylpropanoid biosynthesis in the roots. Overexpressed genes in the lignin biosynthesis in the roots might improve Cd accumulation in cell walls. Moreover, the DEGs were also enriched in photosynthesis in the leaves, transferase activity, oxidoreductase activity, and ABA signal transduction, which might also play roles in reducing Cd toxicity in the plants. All the above, clearly suggest that *T. repens* employ several different mechanisms to protect itself against Cd stress, while the cell wall biosynthesis and GSH metabolism could be considered the most important specific mechanisms for Cd retention in the roots of *T. repens*.

## 1. Introduction

Heavy metal contamination has been a prominent environmental issue worldwide, mainly caused by industrial activities (electroplating, smelting, mining, etc.) and agricultural activities (phosphate fertilizer, agrochemicals, manure application, etc.) [1,2]. Cadmium (Cd) is one of the most universal and toxic heavy metals, due to it being readily absorbed, transported, and accumulated in plants as a non-essential element [3,4,5]. Approximately 30,000 tons of Cd were estimated to be discharged into the atmosphere worldwide every year [4]. Excessive Cd is toxic to plants in physiology and biochemistry aspects, such as inhibiting plants’ seed germination, seedling growth, organs development and damaging the membrane system, the antioxidant enzyme system, photosynthesis efficiency, and the other key biosynthetic pathways [3,6]. Furthermore, Cd cannot be biodegraded, and it can enter the food chain easily of its high mobility, which would cause harm to the health of humans and animals [7,8]. Therefore, it is important to explore further the regulatory mechanisms response of plants to Cd toxicity, which is essential to minimize its risk in the food chain and further facilitate decontamination of Cd-contaminated soils by phytoremediation [9,10].

The strategy of plants under heavy metal stress is determined by many aspects, such as heavy metal concentration, species, rhizosphere environment, and related metabolite level [11]. There are different sensitivities to heavy metal exposure were existed among different plant species. Till now, the response mechanism of various herbaceous species to Cd stress has been widely studied, such as *Galega orientalis* [12], *Solanum nigrum* (*S. nigrum*) [10], *Brassica rapa* [13], *Festuca elata* [14], and *Hibiscus cannabinus* [15]. However, the response mechanism of Cd has not been revealed in all the above species. In general, there are two strategies to resist Cd exposure [16,17]. One strategy is the avoidance mechanism, which tries to reduce Cd migration and avoid heavy metals entering roots [18]. Mycorrhiza and root exudates in the rhizosphere play a significant role to avoid heavy metal stress [19]. The other strategy relies on confinement and detoxification of heavy metals, which is the tolerance mechanism [16]. It reduces Cd toxicity mainly with chelation and antioxidant systems [20,21]. Glutathione (GSH) can combine Cd ions with the extending function of phytochelatins (PCs) synthesis, and the PCs-Cd complex is transported into vacuoles for detoxification, which is an efficient defense strategy in non-hyperaccumulator plants [22]. Reactive oxygen species (ROS) will produce under Cd stress, which can cause severe damage to plants. Malondialdehyde (MDA) is an important index of phytotoxicity [23], which indicated the toxicity level of plants [24]. To mitigate oxidative damage to plants, the enzymatic and non-enzymatic antioxidant systems are employed [25]. The most important enzymes include catalase (CAT), peroxidase (POD), superoxide dismutase (SOD) and glutathione reductase (GR) [25], and ascorbic acid (ASA), GSH is a non-enzyme and reduced GSH the main non-enzymatic antioxidant can be oxidized to glutathione disulfide (GSSG) to scavenge ROS [22,26]. Furthermore, the transporter family also has an important role under Cd stress, such as heavy metal ATPase (HMA), ATP-binding cassette (ABC), natural resistance-associated macrophage protein (Nramp), and cation diffusion facilitator (CDF) [27,28].

*Trifolium repens* (*T. repens*), an important perennial legume, has strong adaptability to different environmental conditions and relatively large biomass [29]. Previous studies reported that it has great potential for phytoremediation because it can accumulate significant amounts of heavy metal ions (Cd^2+^, Pb^2+^, Zn^2+^, and Cr_2_O_7_^2−^) in both roots and shoots without visible damage [2,30,31]. For instance, it can accumulate 55.81 and 90.3 mg/kg Pb in 100 and 500 mg/kg Pb contained soil, respectively [31], and heavy metal ions (Cr_2_O_7_^2−^, Cd^2+^ and Pb^2+^) 19.37–168.74 mg/kg in roots and 10.89–86.53 mg/kg in shoots [2]. The Cd concentrations in the below-ground part of *T. repens* are higher than that of *Bidens pilosa* (*B. pilosa*) and *S. nigrum* after seed plasma treatment, and the mean remediation efficiency of *T. repens* is also the highest among the three cadmium-tolerant plant species [29]. However, the preceding research mainly focused on the exogenous additive (e.g., Nitric oxide), microbial community [31], rhizosphere, and endosphere [2], the mechanism of *T. repens* response to Cd stress remains largely unknown. Since *T. repens* has great potential in phytoremediation, elucidating the molecular mechanism underlying *T. repens* Cd response is essential. In recent years, RNA sequencing is a reliable and economic technology, and transcriptomic has been widely used to reveal differential response mechanisms among plants under Cd stress, such as *Zea mays* (*Z. mays*) [32], *Oryza sativa* (*O. sativa*) [33], *Lolium multiflorum* [34], and *Nicotiana* species [35]. 

In this study, an integrated analysis of physiological and comparative transcriptomics was conducted to investigate the short-term response of *T. repens* to Cd toxicity. The objectives of this study were to (Ⅰ) evaluate the Cd uptake and translocation of *T. repens* under Cd stress; (Ⅱ) investigate the plant physiological response under Cd stress, including oxidative damage (MDA), antioxidant enzymes (APX, SOD, POD, and CAT), non-enzymatic products (soluble protein and chlorophyⅡ); (Ⅲ) identify pivotal Cd responsive differentially expressed genes (DEGs) and their involved essential pathways; (IV) unveil the molecular mechanisms of detoxification and tolerance of *T. repens* response to Cd stress. The present study’s findings will be in favor of novel perspectives of the mechanism involved in Cd tolerance and accumulation in *T. repens*.

## 2. Results

### 2.1. Cd Concentrations in Leaves and Roots

**Figure 1:**
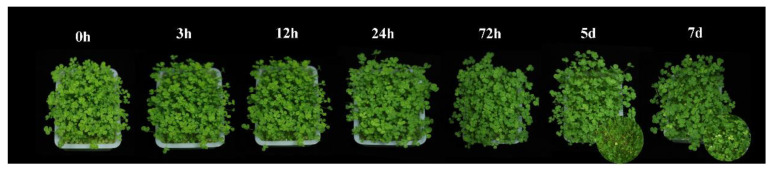
The phenotypic changes of *T. repens* from 0 h to 7 d under the Cd stress.

In the present study, *T. repens* plants were treated with 300 mg/L CdCl_2_·2.5H_2_O solution and the leaves began to turn yellow on the 5th day of treatment (Figure 1). Then, the physiological and transcriptome data were measured in the first 72 h to investigate the coordinated mechanisms of *T. repens* in response to high-level cadmium stress in the early stage. 

**Figure 2:**
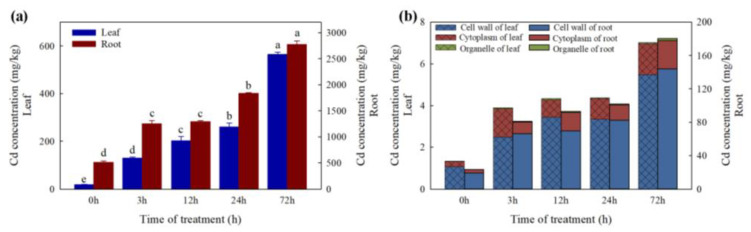
(**a**) Cadmium (Cd) concentrations in roots and leaves, different letters (a–d) in the figure indicate significant differences at *p* < 0.05 level among different time points in the same tissue; (**b**) subcellular distribution of Cd in roots and leaves at 0, 3, 12, 24, and 72 h Cd treatment.

Average leaf Cd concentrations at 0, 3, 12, 24, and 72 h after Cd treatment were 19.82, 129.95, 202.67, 260.56 and 564.72 mg/kg dry weight (DW), and the average root Cd concentration were 511.76, 1249.69, 1294.19, 1838.34 and 2773.95 mg/kg. The translocation factor is always less than 0.2 under Cd treatment in this study. The most dramatic change of Cd content occurred at 3 h after Cd treatment with an increase of 2.44 times in roots and 6.56 times in leaves. Moreover, between 24 h and 72 h, the increase in Cd content was still significant (1.51 times in roots and 2.17 times in leaves) (Figure 2a). Between 63.6% and 82.0% of Cd in leaves and roots was distributed in the cell wall fraction and 17.1–35.2% was distributed in the soluble fraction (cytoplasm). There was only a small amount of Cd (0.6–1.8%) distributed in organelles in *T. repens* (Figure 2b).

### 2.2. Changes in Physiology Characters under the Cd Stress

**Figure 3:**
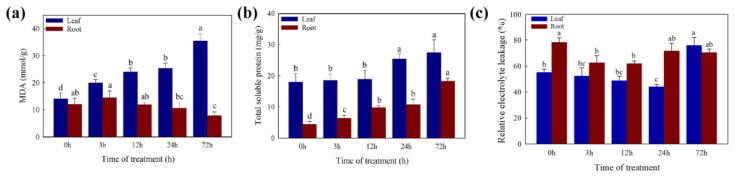
Measurement of (**a**) MDA, (**b**) soluble protein, and (**c**) electrolyte leakage levels. Different letters (a–d) in the figures indicate significant differences at *p* < 0.05 level among different time points in the same tissue.

The MDA, SP, and EL were measured to assess the physiological response of white clover under Cd stress (Figure 3). With the increase in Cd treatment time, the MDA content was significantly increased in leaves and no significant difference in roots. The SP content was significantly increased in leaves and roots with the increase in Cd treatment time. The value of EL first goes down and then goes up during the 72 h treatment period. The EL value decreased significantly after 3 h and 24 h in the roots and leaves, respectively. 

**Figure 4:**
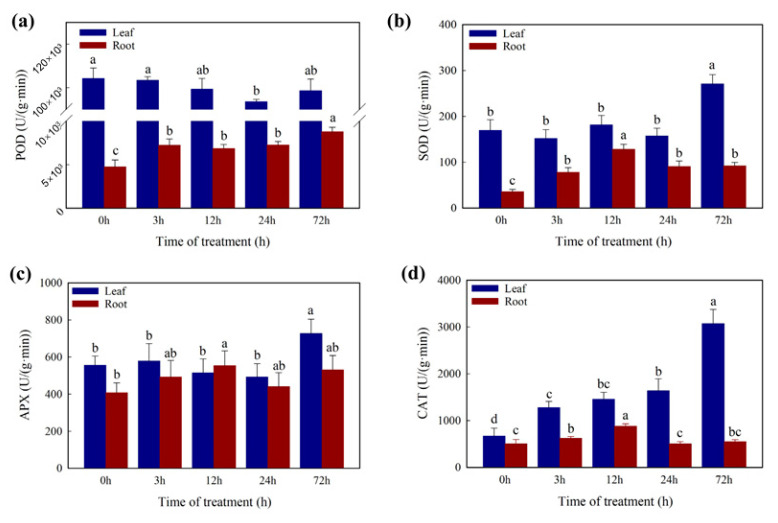
Measurement of antioxidative enzyme activity (**a**) POD activity, (**b**) SOD activity, (**c**) APX activity, (**d**) CAT activity. Different letters (a–d) in the figures indicate significant differences at *p* < 0.05 level among different time points in the same tissue.

In addition, the content of SOD and APX (Figure 4) showed a significant increase after 72 h Cd treatment and no significant difference during the first 24 h in leaves. The CAT content (Figure 4) was increased significantly in the first 3 h and after 72 h Cd treatment in leaves. There was no significant difference in POD (Figure 4) during the 72 h Cd treatment in leaves. In roots, the content of SOD and CAT was increased significantly in the first 12 h, the POD was significantly increased in the first 3 h and the APX showed no significant difference.

### 2.3. Transcriptome Sequencing and DEGs Identification

All the sequencing data were deposited in the National Center for Biotechnology Information (NCBI) Sequence Read Archive (SRA) database (accession no. RJNA771135). A total of 1,355,558,820 raw data was generated from the 30 samples. The raw reads ranged from 40,385,064 to 50,694,506. After the data filtering, the clean reads ranged from 38,718,352 to 48,295,400, and a total of 1,298,978,268 clean reads were generated. The data has a high level of quality, which is indicated by the Q20 and Q30 values, more than 97.89% and 93.90%, respectively (Table S2). The GC content ranged from 40.87% to 41.92%. Of the 30 to 48 million clean reads, 85.04% to 91.78% were mapped to the reference *T. repens* genome, and 75.84% to 79.85% were mapped uniquely to the genome [36] (Table S2). Around 75.06% to 81.04% were mapped to the exons, 3.73% to 4.77% were mapped to the introns, and 15.23% to 20.84% were mapped to the intergenic region (Table S2). 

**Figure 5:**
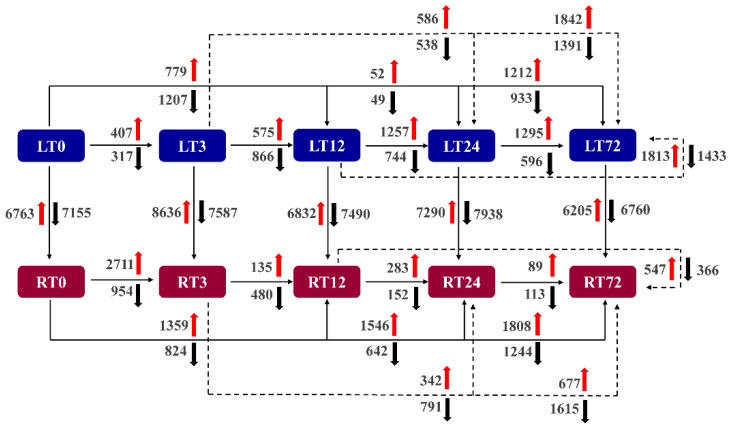
Summary of differentially expressed genes (DEGs) between organs and treatment duration.

To further understand the dynamic expression changes of DEGs in *T. repens* at different time points after Cd treatment in leaves and roots, twenty-five comparison groups were analyzed (Figure 5). In leaves, the most remarkable gene expression change occurred in 72 h compared to 0 h. In the comparison, 2145 genes were altered (LT72 vs. LT0), with 1212 genes up-regulated and 933 genes down-regulated. In comparison with the other time points, the most remarkable changes were also found after 72 h Cd treatments. However, the most remarkable gene expression change (3665 genes altered) occurred during the first 3 h in roots, 2711 of which were up-regulated and 954 of which were down-regulated. Furthermore, a high gene alternation level existed between leaves and roots due to the different organs and complicated transcriptional responses to Cd. In the comparisons between leaves and roots, the DEGs at 3 h have been increased to 16,223 from 13,918 DEGs at 0 h, which indicated that Cd stress in the first 3 h can significantly impact the transcriptional profiles consistent with the above analysis.

In the analysis of the overlaps of DEGs in leaves, there was only one common gene among all the gene sets during the whole-time course (Figure S1). Almost all the Cd-induced genes and Cd-repressed genes in leaves at different gene sets were distinct. There were only 2 common genes between the up-regulated gene sets LT3 vs. LT0 and LT12 vs. LT3, 3 common genes between LT12 vs. LT3 and LT24 vs. LT12, and 4 common genes between LT24 vs. LT12 and LT72 vs. LT24. Among the down-regulated gene sets, there were 17 genes between LT3 vs. LT0 and LT12 vs. LT3, 1 common gene between LT12 vs. LT3 and LT24 vs. LT12, and 1 common gene between LT24 vs. LT12 and LT72 vs. LT24. In roots, there are no common genes among all the data sets, including the up-and down-regulated gene sets. Among the up-regulated gene sets, no common genes were found between RT3 vs. RT0 and RT12 vs. RT3, RT24 vs. RT12, and RT72 vs. RT24. There was only one common gene between up-regulated gene sets RT12 vs. RT3 and RT24 vs. RT12. Among the down-regulated gene sets, there was no common gene between RT12 vs. RT3 and RT24 vs. RT12, RT24 vs. RT12, and RT72 vs. RT24. Moreover, 22 common genes were found between the down-regulated gene sets RT3 vs. RT0 and RT12 vs. RT3. The overlapping genes between leaves and roots were also explored. Among all the data sets, 7385 common genes existed during the whole Cd treatment. There were 3309 common up-regulated genes, and 4071 common down-regulated genes were found among all the gene sets. 

### 2.4. GO, KEGG Enrichment Analysis of DEGs, and WGCNA Analysis

**Figure 6:**
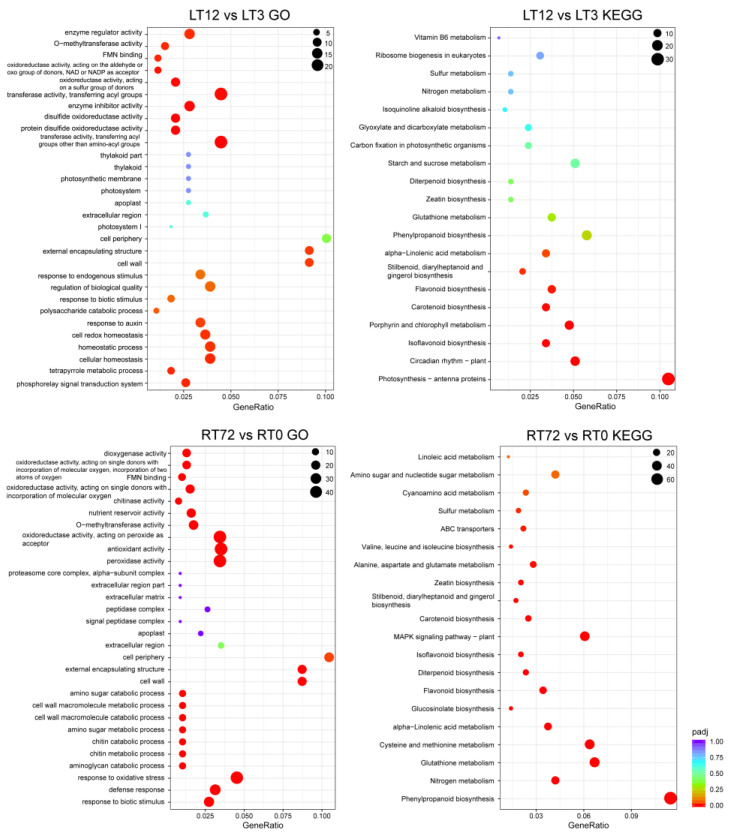
GO and KEGG enrichment analysis of all DEGs following CdCl_2_ exposure: LT12 vs. LT3 in leaves and RT72 vs. RT0 in roots.

The GO and KEGG enrichment analysis of identified DEGs under Cd stress in *T. repens* was conducted (Figure 6 and Figure S2). The results showed that the DEGs in leaves in the first 3 h Cd treatment were enriched in several general metabolic processes, such as alcohol metabolic process, polyol metabolic process, and the organic hydroxy compound metabolic process by GO analysis, and circadian rhythm-plant by KEGG analysis. After 12 h Cd treatment, the DEGs were mainly enriched in transferase activity, disulfide oxidoreductase activity, oxidoreductase activity, response to oxidative stress and response to the hormone by GO analysis, and photosynthesis-antenna proteins, phenylpropanoid biosynthesis, plant-pathogen interaction, MAPK signaling pathway-plant, Cysteine, and methionine metabolism by KEGG analysis (e.g., LT12 vs. LT3). In roots, the DEGs are mainly enriched in transferase activity, antioxidant activity, oxidoreductase activity, peroxidase activity, response to oxidative stress, membrane protein complex, defense response, response to the biotic stimulus by GO analysis, sulfur metabolism, glutathione metabolism, phenylpropanoid biosynthesis, Nitrogen metabolism, and MAPK signaling pathway-plant by KEGG analysis (e.g., RT72 vs. RT0).

**Figure 7:**
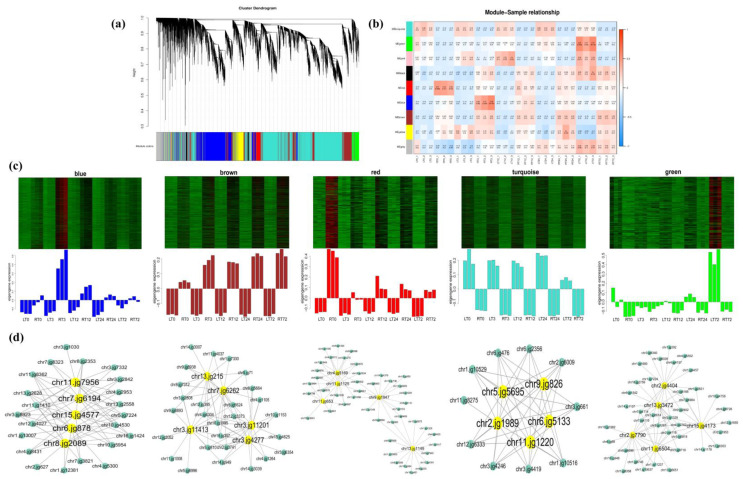
Weighted gene co-expression network (WGCNA) analysis of DEGs of *T. repens* under Cd treatment. (**a**) Cluster dendrogram of 9 different expression modules; (**b**) module-sample relationship; (**c**) expression levels of genes among five selected modules most significantly correlated with roots and leaves under Cd stress; (**d**) the correlation networks of hub genes of the DEGs corresponding to the modules in WGCNA, the Kwithin value was showed in Table S3.

A total of 9 modules were generated of the 38,147 genes from 30 samples (Figure 7) in the WGCNA analysis. Of which, 5 modules were remarkably related to the different tissues (leaves and roots) at multiple time points. The “blue”, “brown”, and “red” modules showed the expression specificity in roots, while the expression specificity of leaves was mainly shown in “turquoise” and “green” modules. In the present study, the top 5 genes with the highest Kwithin value of the identified DEGs under Cd stress in each module were considered as the hub genes in the corresponding pathway (Table S3). In roots, the hub genes are mainly related to the genes of phenylpropanoid biosynthesis in the “blue” module (*chr6.jg878*, *chr11.jg7956*, *chr15.jg4577*, *chr8.jg2089*, and *chr7.jg6194*). In the “brown” module, the hub genes were mainly related to the bZIP transcription factor (*chr3.jg11413*), the antioxidant enzyme (*chr3.jg11201*), and the transporters (*chr7.jg6262*, *chr3.jg4277*, and *chr13.jg215*). The hub genes in the “red” module were related to the bHLH transcription factor (*chr4.jg5169*) and the glutamine metabolism pathway (*chr11.jg653*, *chr13.jg1149*, and *chr11.jg1125*). In leaves, the hub genes were mainly related to the glutamine metabolism pathway (*chr9.jg826*, *chr11.jg1220*, and *chr2.jg1989*), the bHLH transcription factor (*chr5.jg5695*), and the antioxidant enzyme (*chr6.jg5133*) in the “turquoise” module. In the “green” module, the hub genes were mainly related to transporters (*chr13.jg3472*), the process of chlorophyll a/b biosynthesis (*chr2.jg7790* and *chr11.jg6504*), the transcription factor (*chr2.jg4404*), and the ABA signal transduction pathway (*chr15.jg4173*). The co-expression networks of the hub genes of each module were shown in Figure 7d.

### 2.5. DEGs Involved in Lignin Biosynthesis and Heavy Metal Transporters in Cd Stress Response

**Figure 8:**
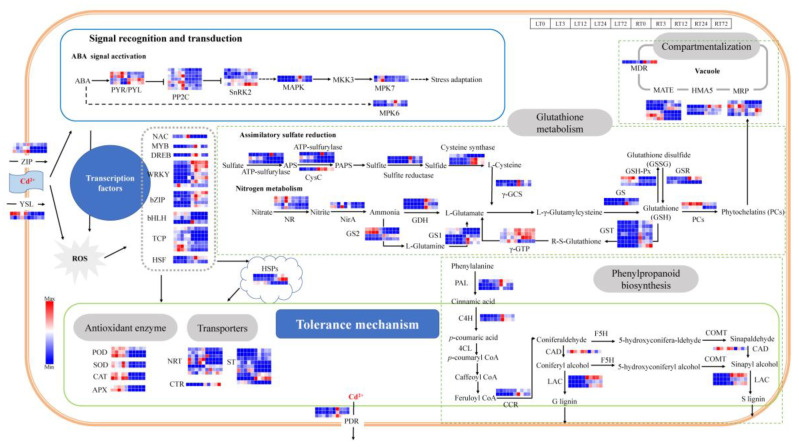
Transcriptional changes of genes responsible for Cd response in leaves and roots of *T. repens*.

The plant cell wall is the first barrier and has a significant fixation effect on heavy metal ions. Lignin is the main component of the cell walls and its biosynthesis can potentially increase the binding efficiency of the cell walls to the heavy metal ions [37]. In the present study, the DEGs were enriched in phenylpropanoid biosynthesis after 3 h and 72 h Cd treatment in roots and 12 h in leaves (Figure 6 and Figure S2), which was the main biosynthesis way of lignin in plants. A total of 11 DEGs were identified which involved in the lignin biosynthesis (Figure 8, Table S3), including 3 DEGs related to phenylalanine ammonia-lyase (PAL), 2 DEGs related to cinnamate 4-hydroxylase (C4H), 2 DEGs related to cinnamoyl-CoA reductase (CCR), 3 DEGs related to laccase (LAC) was induced in roots and leaves under Cd stress. The above DEGs were induced by Cd stress both in leaves and roots, and the highest expression level was shown after 3 h Cd treatment in roots. There was only one DEG related to cinnamyl alcohol dehydrogenase (CAD) that was induced both in leaves and roots, and the expression level was higher in leaves than in roots during 3 to 72 h under Cd treatment.

Furthermore, the Cd ions were absorbed into the plants mainly through the absorption pathway of divalent cations (e.g., Mn^2+^, Fe^2+^, Ca^2+^, Zn^2+^). Lots of transporters participate in the transfer processes of Cd in the plants. In the present study, 40 differentially expressed transport genes were identified which are possibly related to the Cd stress response (Figure 8, Table S3). The DEGs were induced by Cd stress both in leaves and roots. There were 3 DEGs related to Zinc/Iron-regulated transport protein (ZIP), which played an important role in the process of Cd entering into the cells. The Cd could also enter cells through Cd-chelates by yellow stripe 1-like (YSL) proteins (2 DEGs). The expression level of DEGs in leaves was higher than in roots. There were 8 DEGs related to the ATP-binding cassette (ABC) transport superfamily, including 3 DEGs related to pleiotropic drug resistance protein (PDR), 1 DEG related to multidrug resistance protein (MDR), and 5 DEGs related to multidrug resistance-associated protein (MRP). The DEGs expression level of MDR and PDR in roots was higher than in leaves. There were 2 DEGs expression levels related to MRP that was higher in leaves than in roots at all time points (*chr8.jg2040* and *chr3.jg2150*), and the other 2 DEGs had higher expression level in roots (*chr13.jg4938* and *chr13.jg4939*). There were 6 DEGs related to multidrug and toxic compound extrusion protein (MATE), 3 DEGs had higher expression levels in roots (*chr1.jg10735*, *chr9.jg1754*, and *chr7.jg6262*) and the other 3 DEGs had higher expression levels in leaves (*chr4.jg4796*, *chr5.jg4276*, and *chr8.jg1983*). There were 3 DEGs related to Heavy metal transporting ATPase (HMA) and 1 DGE related to Copper transporter (CTR), of which the expression levels were higher in roots. Moreover, there were 8 DEGs related to Nitrate transport protein (NRT) identified, and 5 DEGs had higher expression levels in leaves (*chr13.jg3472*, *chr6.jg100*, *chr13.jg1054*, *chr5.jg1644*, and *chr11.jg4375*) and the other 3 DEGs had higher expression levels in roots. There were 9 DEGs related to Sulfate transporter (ST), and 5 DEGs had higher expression levels in roots (*chr2.jg57*, *chr2.jg8*, *chr7.jg512*, *chr4.jg12030*, and *chr12.jg1561*) and the other 3 DEGs had higher expression levels in leaves. Most of the above genes were significantly expressed with the increase in treatment time. 

### 2.6. DEGs Involved in Oxidation Resistance in Cd Stress Response

A large number of reactive oxygen species (ROS) could be generated under Cd stress, and the oxidation resistance systems in plants can be activated to prevent membrane lipid peroxidation and protect cells through scavenging ROS. The results of the present study identified 58 DEGs that were related to detoxification in white clover in response to Cd stress (Figure 8, Table S3). The antioxidant enzymatic genes, including CAT (2 DEGs), SOD (2 DEGs), POD (3 DEGs), APX (2 DEGs), glutathione reductase (GSR, 2 DEGs), glutathione peroxidase (GSH-Px, 3 DEGs) were induced both in leaves and roots. The GSR-related genes had higher expression levels in roots than in leaves, and the highest expression level was highest after 3 h Cd treatment in roots. Besides the GSR-related genes, the expression levels of the above genes were higher than in leaves.

As an important non-enzymatic antioxidant, Glutathione (GSH) has high efficiency in scavenging free radicals and resisting peroxidative damage in plants. The DEGs in the assimilatory sulfate reduction pathway and nitrogen metabolism pathway related to the GSH precursor biosynthesis have been identified in the study. In the assimilatory sulfate reduction pathway, the ATP-sulfurylase (3 DEGs), CysC (1 DEGs), sulfite reductase (2 DEGs), and cysteine synthase (3 DEGs), were induced in both leaves and roots. The expression level of the above genes was higher in roots and significantly up-regulated at 3 h Cd treatment in roots. In the nitrogen metabolism pathway, nitrate reductase (NR, 4 DEGs), Nitrite reductase (NirA, 2 DEGs), glutamate dehydrogenase (GDH, 3 DEGs), γ-glutamylcysteine synthetase (γ-GCS, 2DEGs), Glutamine synthetase (GS1, 5 DEGs; GS2, 4 DEGs) were induced both in leaves and roots. The expression levels of NR and NirA were higher in leaves and roots, and significantly up-regulated at 24 h Cd treatment in leaves. The GDH related genes have higher expression levels in roots and are significantly up-regulated at 3 h Cd treatment in roots. Four DEGs (*chr2.jg2114*, *chr2.jg1989*, *chr6.jg3383*, and *chr11.jg1220*) of GS had higher expression levels in leaves, and the other 5 DEGs (*chr11.jg653*, *chr11.jg1125*, *chr4.jg11268*, *chr1.jg1885* and *novel.837*) express higher in roots. Furthermore, the glutathione S-transferase-related genes (GST, 7 DEGs) had high expression levels in roots than in leaves and were significantly up-regulated at 3 h and 72 h Cd treatment. The γ-glutamyl transpeptidase-related genes (γ-GTP, 4DEGs) were induced both in leaves and roots, which were expressed higher in roots. The PCs could also be catalyzed by phytochelatin synthase (PCs, 2DEGs). The induction expression of PCs related genes both in roots and leaves.

### 2.7. DEGs in Hormone Signal Transduction and Transcription Factors (TFs) in Cd Stress Response

In terms of signal recognition and transduction, the ABA signal transduction pathway (22 DGEs) was activated in white clover under Cd stress (Figure 8, Table S3). PYR/PYL (4 DEGs) in the pathway was induced both in leaves and roots, and 2 DEGs (*chr8.jg428* and *chr16.jg1328*) were expressed higher in leaves and the other 2 DEGs (*chr13.jg4773* and *chr1.jg10967*) expressed higher in roots. The other members, including PP2Cs (7 DEGs), SnRK2 (5 DEGs), MAPK (2 DEGs), MPK7 (2 DEGs), and MPK6 (DEGs) were also induced under Cd stress.

Moreover, the plant hormone signaling molecules could also impact the expression of transcription factors (TFs) and other defense proteins under the Cd stress. In the present study, a total of 27 DEGs were identified as TFs in *T. repens* under Cd stress (Figure 8, Table S3). The identified TF families include NAC (1 DEG), MYB (1 DEG), DREB (1 DEG), WRKY (7 DEGs), bZIP (5 DEGs), bHLH (3 DEGs), TCP (7 DEGs), and heat shock transcription factor (HSF, 2DEGs). The above genes were induced both in leaves and roots. The NAC TF family was significantly up-regulated at 72 h Cd treatment in leaves. The MYB and DREB TF families were up-regulated at 3 h Cd treatment in roots. Most of the genes of WRKY and bZIP TF families had higher expression levels in roots. Four DEGs (*chr9.jg5416*, *chr7.jg7338*, *chr16.jg4775*, and *chr4.jg5478*) of the WRKY TF family were significantly up-regulated at 3 h in roots, 2 DEGs (*chr1.jg12558* and *chr7.jg1916*) at 72 h in roots, and 1 DEG (*chr7.jg4527*) at 3 h in leaves. Among the genes related to bZIP, 1 DEG (*chr3.jg11413*) was significantly up-regulated at 72 h and the other DEGs at 3 h Cd treatment in roots. The HSF TF family were significantly up-regulated at 3 h Cd treatment in roots, and most of the genes encoding HSP had higher expression level in roots.

### 2.8. Changes in Photosynthesis under Cd Stress

**Figure 9:**
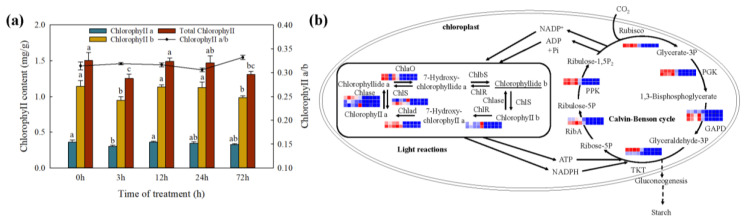
The change of (**a**) chlorophyll contents, and different letters (a, b, c) in the figures indicate significant differences at *p* < 0.05 level among different time points in the same parameter; (**b**) the expression levels of the photosynthetic related genes under Cd stress.

Furthermore, the total Chlorophyll content (Figure 9), Chlorophyll a, and Chlorophyll b were decreased significantly in the first 3 h and no significant difference in the subsequent processing time. The result of the KEGG enrichment analysis showed the DEGs were enriched in photosynthesis-antenna proteins at 12 h Cd treatment in leaves. A total of 25 DEGs were identified, including 11 DEGs in light reactions, and 14 DEGs in the Calvin-Benson cycle (Figure 9, Table S3). In the process of chlorophyll a/b biosynthesis and photosynthetic carbohydrate metabolism, the DEGs were down-regulated as the Cd treatment increased in leaves. The expression levels of the DEGs were extremely low in roots.

### 2.9. Validation of Transcriptome Data by qRT-PCR Analyses

**Figure 10:**
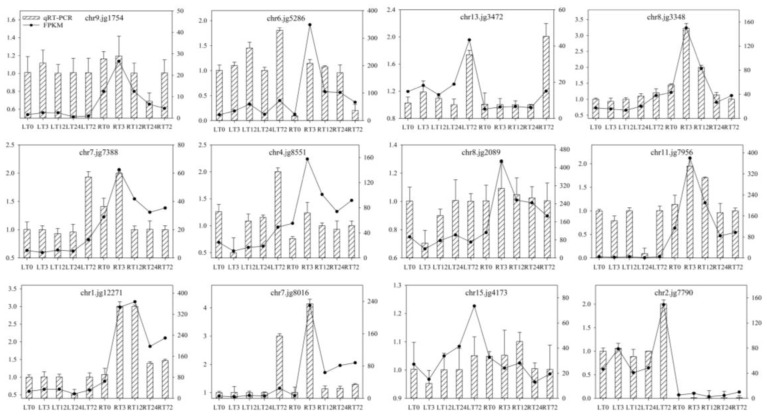
Expression of the selected 12 genes inferred at 0, 3, 12, 24, and 72 h by RNA-seq and qRT-PCR.

To confirm the RNA-seq results, 12 genes were randomly selected for qRT-PCR, including genes related to transporter protein, transcription factor, lignin synthesis, glutathione metabolism, hormone signaling, and chlorophyll biosynthesis (Table S1). Overall, the relative gene expression patterns of qRT-PCR were consistent with the RNA-seq (Figure 10) at all the treatment points in the roots and leaves of *T. repens*. The results showed that the RNA-seq result was reliable in the present study.

## 3. Discussion

Based on the responses to the heavy metals, the plants can be divided into four types: metal-sensitive species, metal-tolerant nonhyperaccumulator species, metal-resistant excluder species, and metal hyper tolerant hyper-accumulator species [35]. *T. repens* could be classified into metal-tolerant species for it could grow well in metal-contaminated soils and has a significant accumulation of Cd, Cr, and Pb without visible damage [2,29,31,38]. The plant growth condition did not be affected by low concentration heavy metals, and the heavy metal accumulation ability of *T. repens* was significantly higher at a high background heavy metal concentration [2]. Moreover, the heavy metal accumulation ability does not appear to be limited, since the Cd concentration in plant tissues continues to rise with increasing exposure time [38]. Moreover, the highest Cd concentration 578 mg/kg has been found in China [39]. Previous studies [39] also found that the Cd concentration exceed the limit value (1 mg/kg) at about a quarter of the surveyed sites in China. Therefore, it is very worthwhile to study the tolerance of *T. repens* under high cadmium concentration in this study.

*T. repens* can accumulate 40 mg/kg in roots and no significant Cd in shoots after 3 days of exposure in the soil contaminated with 20 mg/kg. Moreover, for 56 days of exposure, the Cd concentration could be reached 106 and 3.8 mg/kg, in roots and shoots, respectively [38]. Moreover, the *T. repens* can accumulate Cd as high as 25.6 mg/kg (shoots) and 45.4 mg/kg (roots) after cold plasma seed treatment under the background Cd content is 3.02 mg/kg [29], while the Cd concentrations in the roots were even higher than two Cd hyperaccumulator, *S. nigrum* [40] and *B. Pilosa* [41]. In the current study, the results showed a high accumulation of Cd in the leaves and roots (Figure 1) under the high-level background Cd content and show no significant negative symptoms for 72 h exposure. The results showed the higher accumulation may be attributed to the quartz sand culture, which is similar to the results of *Salvia sclarea* (*S. sclarea*) [42], *Malva parviflora* [43], and *Noccaea caerulescens* [44] in hydroponic solution. Furthermore, the translocation factor is always less than 0.2 in our study, which is consistent with the previous study [38]. Besides, *T. repens* can also adapt to wide conditions with an extensive roots system and a large amount of biomass suggesting that *T. repens* is a candidate plant for Cd phytoextraction and phytostabilization [42].

As the first natural barrier of plant cells, the cell wall shows a significant fixation effect on heavy metal ions in keeping excess Cd out of the cell [37,45]. In our study, most of the Cd was bound to the plant cell wall and cytoplasm (Figure 1) and has a significant proportion in the cell wall. The results are consistent with that of *Z. mays*, *Boehmeria nivea*, *Medicago sativa*, and *Nicotiana rustica*, while the Cd was mainly accumulated in the soluble component in *O. sativa*, *Hordeum vulgare*, *Phytolacca americana* (*P. americana*), *Amaranthus hypochondriacus*, and *Nicotiana tabacum* [35,46]. The plant cell wall biosynthesis also is enhanced under the Cd stress to improve the Cd tolerant ability [47]. In our study, KEGG analysis showed that phenylpropanoid biosynthesis was over-presented in white clover under Cd exposure. The genes identified in the WGCNA are also hubs in phenylpropanoid biosynthesis. It is an important defense strategy for plants and can generate various metabolites, including guaiacyl and syringyl lignin, which are essential for cell wall biosynthesis [48,49]. In our study, 8 DEGs (PAL, C4H, CCR, and LAC) were up-regulated in roots related to lignin biosynthesis and achieved the highest expression level after 3 h Cd treatment. Increased lignin synthesis in roots to block Cd into the plant cells has also been identified in *Vicia sativa* [50], *Brassica chinensis* (*B. chinensis*) [47], *Solanum melongena* [51], *Nicotiana* species [52], and *S. nigrum* [10] under Cd stress. In addition, POD could regulate the biosynthesis of lignin and the related genes were upregulated in our studies [37,48]. The results indicated that the lignification biosynthesis would be strengthened once expose to Cd stress and improve the Cd fixed ability of the cell wall in *T. repens*. 

Elevated ROS generation under Cd stress would disrupt the plant cell membranes with oxidative injuries by promoting the lipid peroxidation of membranes [53,54]. The amounts of MDA, EL, and SP were reported as the criteria for the identification of heavy metal tolerance [55]. However, the three indicators showed a strong increasing pattern in general, which showed that the damage to the membrane caused by Cd became more serious with the Cd treatment time increasing. Moreover, then the antioxidant defense system (POD, SOD, CAT, APX, etc.) was activated to mitigate H_2_O_2_ and lipid peroxidation damage [56,57]. For this study, the amounts of the antioxidant enzymes (POD, SOD, CAT, and APX) also showed an increasing pattern in general, besides the amount of CAT showed a decreasing pattern in the roots. The results suggest that the generated ROS might overwhelm the defense ability of the CAT enzyme in roots during the 72 h Cd treatment in *T. repens*. Furthermore, most of the related genes were up-regulated significantly in the leaves of *T. repens*, which were beneficial to remove excess ROS [35,53]. The HSP was also found to play a significant role in protecting the plant from adverse environments [45,58], and the expression level of HSP genes was also found up-regulated under Cd stress in *T. repens*. 

GSH plays an important role in the detoxification of Cd in plants, which is related to both the chelation of Cd and scavenging ROS [59]. On the other hand, GSH is the precursor of PCs, and the PCs can bind most heavy metal ions [60]. It has been proven that the increased synthesis of GSH and PCs improves Cd tolerance in rice [61]. The expression level of PCs encoding genes was found up-regulated in *T. repens* (Figure 8) and then promoted the Cd accumulation in plants [37]. In the present study, KEGG analysis showed that the DEGs were enriched in sulfur metabolism, glutathione metabolism, and Nitrogen metabolism. The genes identified in the WGCNA are also hubs in glutathione metabolism. The DEGs related to the ATP sulfurylase, and cysteine synthase of the sulfur metabolism pathway were up-regulated both in the roots and leaves of *T. repens*. They are key enzymes for cysteine biosynthesis, which is the precursor for GSH biosynthesis [62]. Meanwhile, the genes related to NR, NirA, GS and GDH, and γ-GCS were also up-regulated under Cd stress in *T. repens*, which is consistent with the results of *B. chinensis* [63], *Setaria italica* [64], *O. sativa* [33]. In the glutathione metabolism, the DEGs related to GST were up-regulated in the roots of *T. repens*, which have unique functions in Cd detoxification [65]. The expression levels of GST genes were also up-regulated in *O. sativa* [66], *Nicotiana* species [35], and *P. americana* [67]. GST was able to catalyze GSH to be conjugated with ROS to protect plant cells from oxidative damage [68]. GST could catalyze the covalent binding of the cytotoxic substrates with GSH, and then formed S-glutathione conjugated which will be transferred into vacuolar to sequestrate the Cd [69].

The heavy metal ion transporters located on the plant membranes and the tonoplast take an important role in the transport and uptake of Cd [67]. The ABC transport family mainly (PDR, MDR, and MRP) is located in the tonoplast and can transport Cd conjugated complexes as Cd pumps [27,37]. In the current study, the ABC transporters related genes showed up-regulated after 3 h Cd treatment in *T. repens*. The up-regulated MRP and MDR genes can increase the transport of Cd amount into the vacuoles both in the leaves and roots, thereby reducing the Cd toxicity to the plants [70]. The up-regulated PDR genes can promote the Cd ion extrusion and improve the Cd resistance of *T. repens*, which is similar in *Arabidopsis thaliana* (*A. thaliana*) [71], *Arachis hypogaea* [72], and *Lycopersicon esculentum* [45]. We also identified 6 genes encoding MATE transporters up-regulated in *T. repens*, mainly transporting the exogenous toxic substances [73], which were consistent with the study on *O. sativa* [74] and *Festuca arundinacea* [14]. The YSL and ZIP genes were also up-regulated, which indicated the higher capacity of Cd^2+^ uptake and lower Cd symplastic loading into xylem in *T. repens* [52].

The response to high-dose Cd exposure could be ascribed to the plant signal perception and transduction [75,76] and reduction of photosynthetic carbon assimilation [77]. The calcium ion, ABA signal conduction pathway, and mitogen-activated protein kinase (MAPK) pathway were identified as the main signal perception pathway in response to stress tolerance of plants [28,75]. In this study, the member PYR/PYL and PP2Cs of the ABA signal transduction pathway were activated under Cd stress in *T. repens*. Moreover, then the plant hormone signaling molecules can be transmitted through their induced protein, which impacted the related gene regulation by modulating the expression level of TFs or defense proteins. NAC, MYB, DREB, WRKY, bZIP, bHLH, and TCP were identified as up/down-regulated under Cd stress in *T. repens*. Among them, the number of WRKY and TCP genes was the most, and their function in response to Cd stress has been verified in *O. sativa* [33] and *A. thaliana* [78]. The regulation of bHLH and bZIP can improve the Cd tolerance ability [79]. Only one gene was identified related to the other TFs, which showed they were also associated with the stress response in *T. repens* [28] NAC. Meanwhile, the thylakoid membranes of chloroplast would be dissolved under Cd stress, and the chlorophyll synthesis would be inhibited by destroying the chlorophyll photosynthesis enzyme activity [43,54,80]. In our study, the photosynthesis parameters (chlorophyll and chlorophyll a/b) decreased after 3 h of Cd treatment and resumes after 12 h. Except for the chlorophyll, the other two parameters continued to decrease after 72 h, which was also found in soybean plants [81] and *S. sclarea* [42]. The expression level of chlorophyll photosynthesis-related genes was down-regulated after 3 h Cd treatment and began to resume after 12 h. In the study of *Brassica juncea* [82] and *P. americana* [67], the expression level of photosynthesis-related genes decreased rapidly after 12 h of Cd treatment and began to restore after 48 h. The shorter time of this change in *T. repens* may be due to the high concentration of cadmium treated in this study. Taken together, our study contributes to the understanding of the molecular response mechanism in *T. repens* under high concentration Cd stress (Figure 8). At first, Cd caused oxidative stress to cells with excess ROS and induced the ABA hormone signaling pathway. The hormone signaling molecules can be transmitted through the induced protein, thus impacting the expression level of transcription factors (e.g., WRKY, bZIP, bHLH, HSF, etc.) and the other defense proteins [37]. Subsequently, the comprehensive defense system against Cd stress in *T. repens* was activated, including lignification biosynthesis, antioxidant enzyme system, chelation, and vacuolar compartmentalization. The Cd^2+^ was preliminarily blocked in the plant cell walls resulting in the alleviation of Cd toxicity. In the cells, the Cd^2+^ could be combined with various chelating substances (e.g., GSH and PCs) and further compartmentalized in vacuoles. Moreover, the GSH and other antioxidant enzymes would contribute to quenching ROS and protect cells from oxidative damage. In conclusion, *T. repens* showed strong tolerant capacity at a high Cd concentration and could be inferred as a Cd hyper-tolerant plant. Two major tolerance mechanisms under Cd exposure were identified: (1) enhanced the cell wall biosynthesis through the up-regulated genes of phenylpropanoid biosynthesis pathway to alleviate Cd toxicity; (2) enhanced GSH metabolism involved in Cd chelation and induction of antioxidant system against ROS accumulation. The results contribute to the understanding of the molecular physiological response mechanism of Cd stress in *T. repens* which will provide a profound theoretical basis for phytoremediation of Cd pollution. Moreover, a large number of candidate genes were provided in this study, which can be used to further investigate the molecular mechanism of plant tolerance to Cd.

## 4. Materials and Methods

### 4.1. Plant Material and Cd Treatments

The *T. repens* seeds (*cv. Haifa*) were sterilized in 5% sodium hypochlorite solution for 20 and rinsed several times with deionized water. Quartz sand was washed with tap water and deionized water thoroughly and then dried in the dry-heat oven at 65 °C; 0.2 g *T. repens* seeds per pot (20 cm × 15 cm × 12 cm) with 400 g quartz sand was sown. Seedling of *T. repens* was cultivated in a plant growth chamber (25 °C, 16 h light/8 h dark cycle) and irrigated with 1/2 strength Hoagland’s solution, the place of test posts was rotated every day.

After pre-test screening and analysis, *T. repens* was found to have a high tolerance to Cd. Because of this, the plants were treated with 0 mg/L and 300 mg/L CdCl_2_·2.5H_2_O solution 30 days after germination. Three replicates for each organ type (leaf and root) and time point (0 h, 3 h, 12 h, 24 h, and 72 h) were harvested after applying the Cd treatments. The samples were treated with liquid nitrogen immediately and stored at −80° for further analysis.

### 4.2. Cd Concentration and Subcellular Distribution in Leaves and Roots

The Cd concentration in leaves and roots was extracted with concentrated HNO_3_/HClO_4_ (4:1, *v*/*v*), and measured by an Inductively Coupled Plasma Optical Emission Spectrometer (ICP-OES, Optima 8300, CETAC Technologies^®^, Omaha, NE, USA) [83] according to the manufacturer’s recommendations. Cd translocation factor (TF) was defined as the ratio of Cd concentration in the leaves to that in the roots [5]. To determine the subcellular distribution of Cd in leaves and roots, the subcellular components were separated according to previous methods [33]. The Cd concentration of three isolated subcellular components, cell wall, organelle, and cytoplasm, was measured by ICP-OES. Statistical significance analysis was conducted with One-way Analysis of Variance (ANOVA) and the least significant difference (LSD) tests were conducted with SPSS 13.0 (SPSS Inc., Chicago, IL, USA). 

### 4.3. Determination of Physiological Indexes

SOD activity was measured by the nitroblue tetrazole (NBT) method at 560 nm [84]. CAT activity was determined by the hydrolysis of the H_2_O_2_ method at 240 nm [85]. POD activity was investigated by guaiacol as a matrix according to [86] with some modifications at 470 nm [83]. APX activity was determined according to the method of previous studies [87]. The soluble protein (SP) was measured by the Coomassie brilliant blue method [88]. MDA content was measured with the thiobarbituric acid (TBA) method [89]. The electrolyte leakage (EL) was measured [42] with a conductivity meter (DDS-307, REX, Shanghai, China). The contents of photosynthetic pigments, total Chlorophyll content, Chlorophyll a, and Chlorophyll b, in fresh leaves were determined according to Arnon [90]. 

### 4.4. RNA Extraction, Transcriptome Sequencing, and Quality Control

Total RNA was extracted using the RNA prep Pure Plant Plus Kit (Polysaccharides & Polyphenolics-rich) (Tiangen, Beijing, China). RNA integrity was assessed using the RNA Nano 6000 Assay Kit of the Bioanalyzer 2100 system (Agilent Technologies, Santa Clara, CA, USA).

Total RNA was used as input material for the RNA sample preparations. Briefly, mRNA was purified from total RNA using poly-T oligo-attached magnetic beads. Fragmentation was carried out using divalent cations under elevated temperature in First Strand Synthesis Reaction Buffer (5×). First-strand cDNA was synthesized using random hexamer primer and M-MuLV Reverse Transcriptase, then use RNaseH to degrade the RNA. Second strand cDNA synthesis was subsequently performed using DNA polymerase I and dNTP. The remaining overhangs were converted into blunt ends via exonuclease/polymerase activities. After adenylation of 3′ ends of DNA fragments, Adaptor with a hairpin loop structure was ligated to prepare for hybridization. To select cDNA fragments of preferentially 370–420 bp in length, the library fragments were purified with the AMPure XP system (Beckman Coulter, Brea, CA, USA). Then PCR was performed with Phusion High-Fidelity DNA polymerase, Universal PCR primers, and Index (X) Primer. At last, PCR products were purified (AMPure XP system, Beckman Coulter, Brea, CA, USA) and library quality was assessed on the Agilent Bioanalyzer 2100 system. The clustering of the index-coded samples was performed on a cBot Cluster Generation System using TruSeq PE Cluster Kit v3-cBot-HS (Illumia, San Diego, CA, USA) according to the manufacturer’s instructions. After cluster generation, the library preparations were sequenced on an Illumina Novaseq platform and 150 bp paired-end reads were generated (Novogene Co., Ltd. Beijing, China).

Raw data (raw reads) of fastq format were first processed through in-house Perl scripts. In this step, clean data (clean reads) were obtained by removing reads containing adapter, reads containing N base and low-quality reads from raw data. At the same time, Q20, Q30, and GC content of the clean data were calculated. All the downstream analyses were based on clean data with high quality. The clean reads were aligned to the *T. repens* reference genome [36] using HISAT software [91]. 

### 4.5. DEGs, GO, KEGG, and WGCNA Analysis 

Differential expression analysis was performed using the DESeq2 R package (1.20.0) and FPKM (fragments per kilobase of transcripts sequence per million base pairs sequenced) method [92]. The resulting *p*-values were adjusted using Benjamini and Hochberg’s approach for controlling the false discovery rate. Genes with Padj < 0.05 and |log2FoldChange| > 2 were assigned as DEGs (differentially expressed genes) in the present study. 

GO (Gene Ontology) enrichment and KEGG (Kyoto Encyclopedia of Genes and Genomes) pathway analysis were implemented using the cluster profile R package to test the statistical (Padj < 0.05) enrichment of differential expression genes.

In addition, Weighted correlation network analysis (WGCNA) was conducted by R package WGCNA, which can be used for network construction, gene screening, gene cluster identification, topological feature calculation, data simulation, and visualization. Then, the networks were visualized by Cytoscape (v3.6.1).

### 4.6. Gene Expression Validation by qRT-PCR

To evaluate the reliability of the RNA-seq data, 12 candidate genes were selected for validation by quantitative real-time PCR (qRT-PCR) (Table S1). The RNA samples for RNA-seq experiments were also used for qRT-PCR. The primers were designed by the Primer-BLAST (https://www.ncbi.nlm.nih.gov/tools/primer-blast/, accessed on 25 February 2022) and listed in Table S1. Trβ-Actin gene was used as the internal control to quantify the gene expression level in white clover [93]. The first-strand cDNA was synthesized using All-in-One First-Strand Synthesis MasterMix (with dsDNase) (Yugong Biolabs Co., Ltd., Jiangsu, China). qRT-PCR was conducted by using Taq SYBR^®^ Green qPCR Premix (Universal) (Yugong Biolabs Co., Ltd., Jiangsu, China) and performed at Bio-Rad CFX96 real-time PCR detection system. Each sample and gene had three technical replicates and the relative expression levels were evaluated using the 2^−ΔΔCT^ method [94]. 

## Data Availability

The original sequencing data generated in the study have been deposited into the National Center for Biotechnology Information (NCBI) Sequence Read Archive (SRA) database with the accession number RJNA771135.

## Supplementary Materials

The following supporting information can be downloaded at: https://www.mdpi.com/article/10.3390/ijms23094612/s1.
