# Supplementary material for: Unraveling Cadmium Toxicity in Trifolium repens L. Seedling: Insight into Regulatory Mechanisms Using Comparative Transcriptomics Combined with Physiological Analyses
Source: Int J Mol Sci. 2022 Apr 21;23(9):4612. doi: 10.3390/ijms23094612 (PMC9105629; doi:10.3390/ijms23094612)
Supplement: Supplementary file 1 [file ijms-23-04612-s001.zip › ijms-1682701-supplementary/Figure S2. KEGG_GO.pdf]

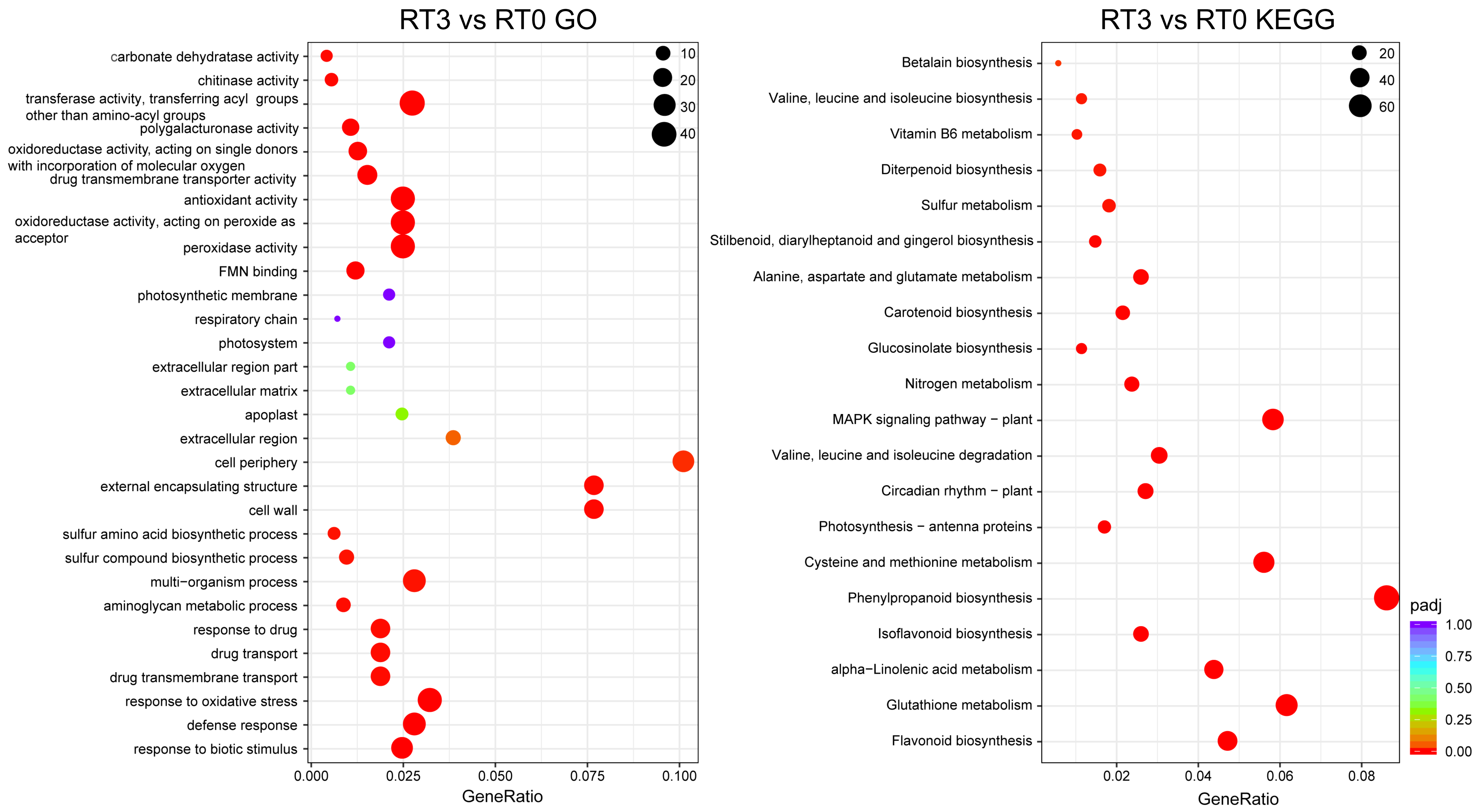

RT3 vs RT0 GO
carbonate dehydratase activity
chitinase activity
transferase activity, transferring acyl groups other than amino-acyl groups
polygalacturonase activity
oxidoreductase activity, acting on single donors with incorporation of molecular oxygen
drug transmembrane transporter activity
antioxidant activity
oxidoreductase activity, acting on peroxide as acceptor
peroxidase activity
FMN binding
photosynthetic membrane
respiratory chain
photosystem
extracellular region part
extracellular matrix
apoplast
extracellular region
cell periphery
external encapsulating structure
cell wall
sulfur amino acid biosynthetic process
sulfur compound biosynthetic process
multi−organism process
aminoglycan metabolic process
response to drug
drug transport
drug transmembrane transport
response to oxidative stress
defense response
response to biotic stimulus
10
20
30
40
0.000
0.025
0.050
0.075
0.100
GeneRatio
RT3 vs RT0 KEGG
Betalain biosynthesis
Valine, leucine and isoleucine biosynthesis
Vitamin B6 metabolism
Diterpenoid biosynthesis
Sulfur metabolism
Stilbenoid, diarylheptanoid and gingerol biosynthesis
Alanine, aspartate and glutamate metabolism
Carotenoid biosynthesis
Glucosinolate biosynthesis
Nitrogen metabolism
MAPK signaling pathway − plant
Valine, leucine and isoleucine degradation
Circadian rhythm − plant
Photosynthesis − antenna proteins
Cysteine and methionine metabolism
Phenylpropanoid biosynthesis
Isoflavonoid biosynthesis
alpha−Linolenic acid metabolism
Glutathione metabolism
Flavonoid biosynthesis
20
40
60
padj
1.00
0.75
0.50
0.25
0.00
0.02
0.04
0.06
0.08
GeneRatio

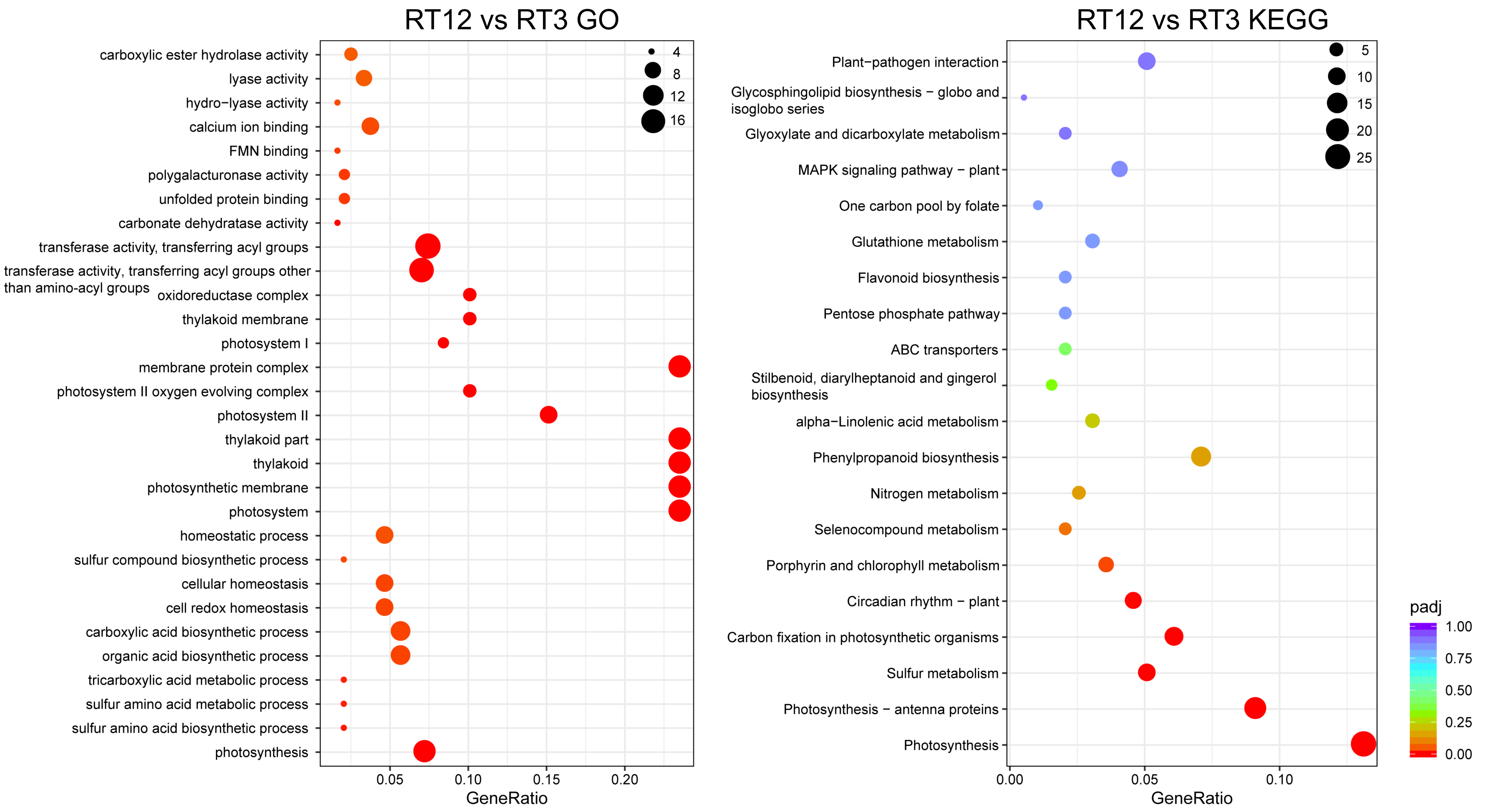
RT12 vs RT3 GO
carboxylic ester hydrolase activity
lyase activity
hydro−lyase activity
calcium ion binding
FMN binding
polygalacturonase activity
unfolded protein binding
carbonate dehydratase activity
transferase activity, transferring acyl groups
transferase activity, transferring acyl groups other than amino-acyl groups
oxidoreductase complex
thylakoid membrane
photosystem I
membrane protein complex
photosystem II oxygen evolving complex
photosystem II
thylakoid part
thylakoid
photosynthetic membrane
photosystem
homeostatic process
sulfur compound biosynthetic process
cellular homeostasis
cell redox homeostasis
carboxylic acid biosynthetic process
organic acid biosynthetic process
tricarboxylic acid metabolic process
sulfur amino acid metabolic process
sulfur amino acid biosynthetic process
photosynthesis
4
8
12
16
0.05
0.10
0.15
0.20
GeneRatio
RT12 vs RT3 KEGG
Plant−pathogen interaction
Glycosphingolipid biosynthesis − globo and isoglobo series
Glyoxylate and dicarboxylate metabolism
MAPK signaling pathway − plant
One carbon pool by folate
Glutathione metabolism
Flavonoid biosynthesis
Pentose phosphate pathway
ABC transporters
Stilbenoid, diarylheptanoid and gingerol biosynthesis
alpha−Linolenic acid metabolism
Phenylpropanoid biosynthesis
Nitrogen metabolism
Selenocompound metabolism
Porphyrin and chlorophyll metabolism
Circadian rhythm − plant
Carbon fixation in photosynthetic organisms
Sulfur metabolism
Photosynthesis − antenna proteins
Photosynthesis
5
10
15
20
25
padj
1.00
0.75
0.50
0.25
0.00
0.00
0.05
0.10
GeneRatio

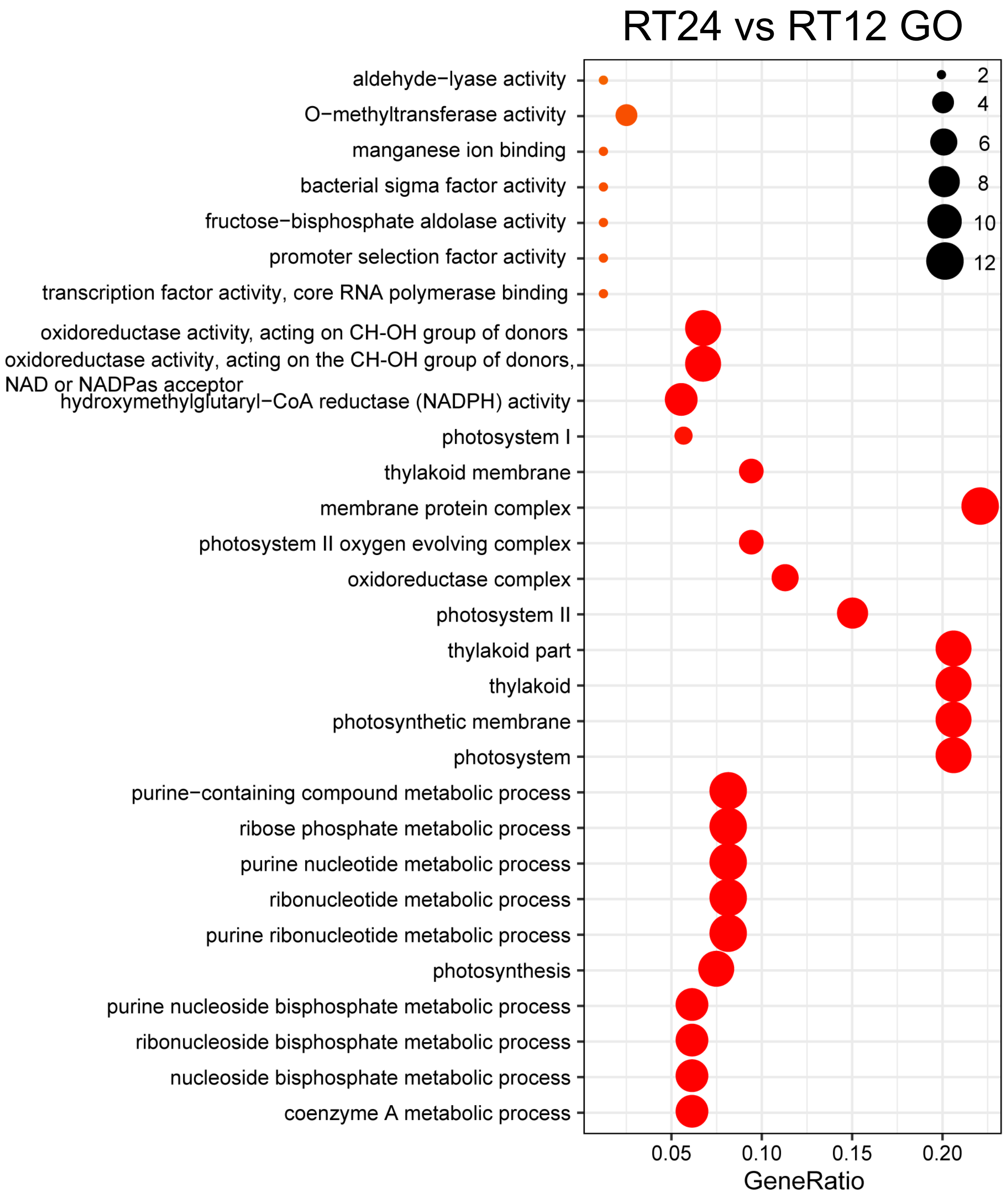
RT24 vs RT12 GO
aldehyde−lyase activity
O−methyltransferase activity
manganese ion binding
bacterial sigma factor activity
fructose−bisphosphate aldolase activity
promoter selection factor activity
transcription factor activity, core RNA polymerase binding
oxidoreductase activity, acting on CH-OH group of donors
oxidoreductase activity, acting on the CH-OH group of donors, NAD or NADPas acceptor
hydroxymethylglutaryl−CoA reductase (NADPH) activity
photosystem I
thylakoid membrane
membrane protein complex
photosystem II oxygen evolving complex
oxidoreductase complex
photosystem II
thylakoid part
thylakoid
photosynthetic membrane
photosystem
purine−containing compound metabolic process
ribose phosphate metabolic process
purine nucleotide metabolic process
ribonucleotide metabolic process
purine ribonucleotide metabolic process
photosynthesis
purine nucleoside bisphosphate metabolic process
ribonucleoside bisphosphate metabolic process
nucleoside bisphosphate metabolic process
coenzyme A metabolic process
2
4
6
8
10
12
0.05
0.10
0.15
0.20
GeneRatio

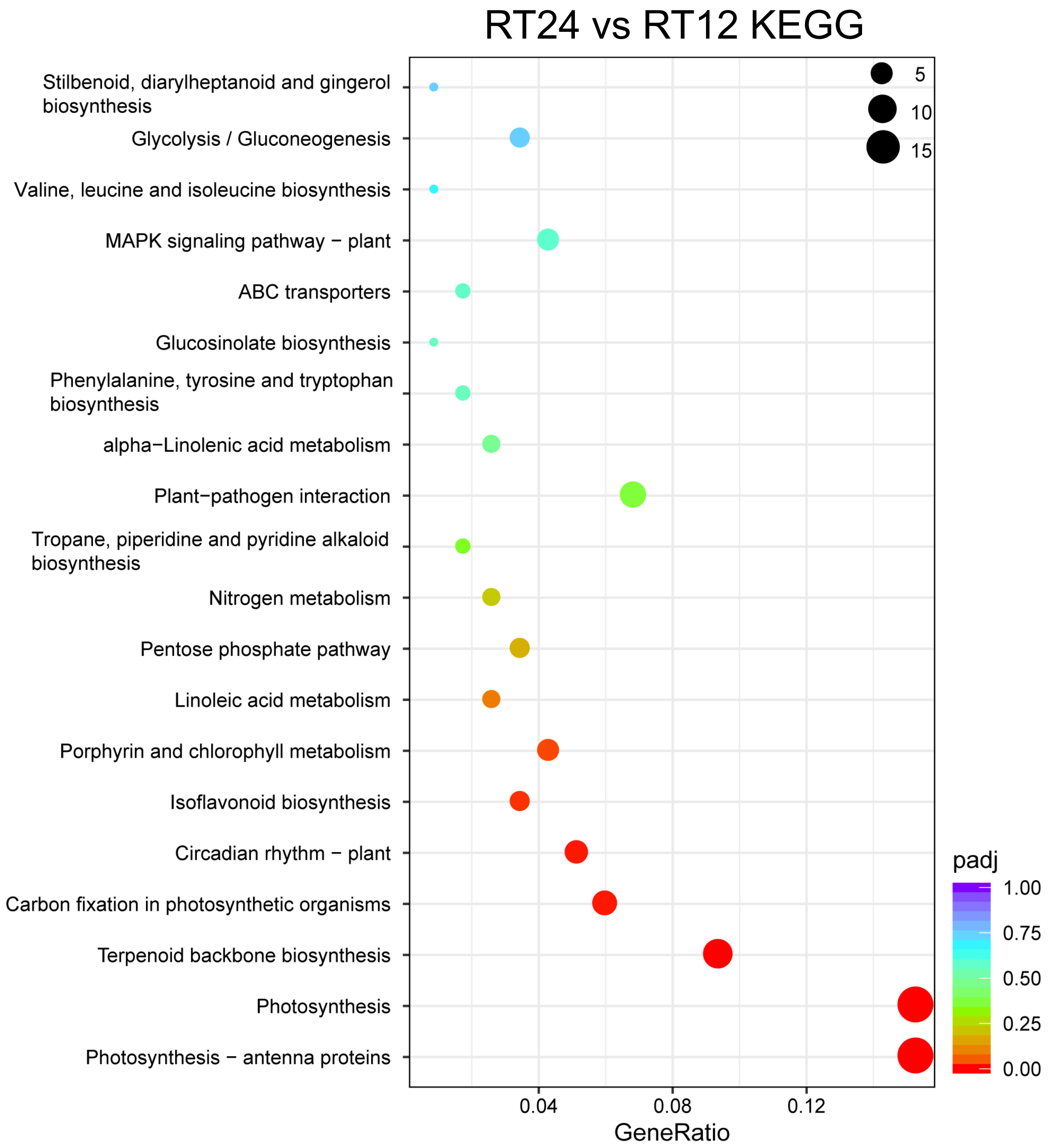
RT24 vs RT12 KEGG
Stilbenoid, diarylheptanoid and gingerol biosynthesis
Glycolysis / Gluconeogenesis
Valine, leucine and isoleucine biosynthesis
MAPK signaling pathway − plant
ABC transporters
Glucosinolate biosynthesis
Phenylalanine, tyrosine and tryptophan biosynthesis
alpha−Linolenic acid metabolism
Plant−pathogen interaction
Tropane, piperidine and pyridine alkaloid biosynthesis
Nitrogen metabolism
Pentose phosphate pathway
Linoleic acid metabolism
Porphyrin and chlorophyll metabolism
Isoflavonoid biosynthesis
Circadian rhythm − plant
Carbon fixation in photosynthetic organisms
Terpenoid backbone biosynthesis
Photosynthesis
Photosynthesis − antenna proteins
5
10
15
padj
1.00
0.75
0.50
0.25
0.00
0.04
0.08
0.12
GeneRatio

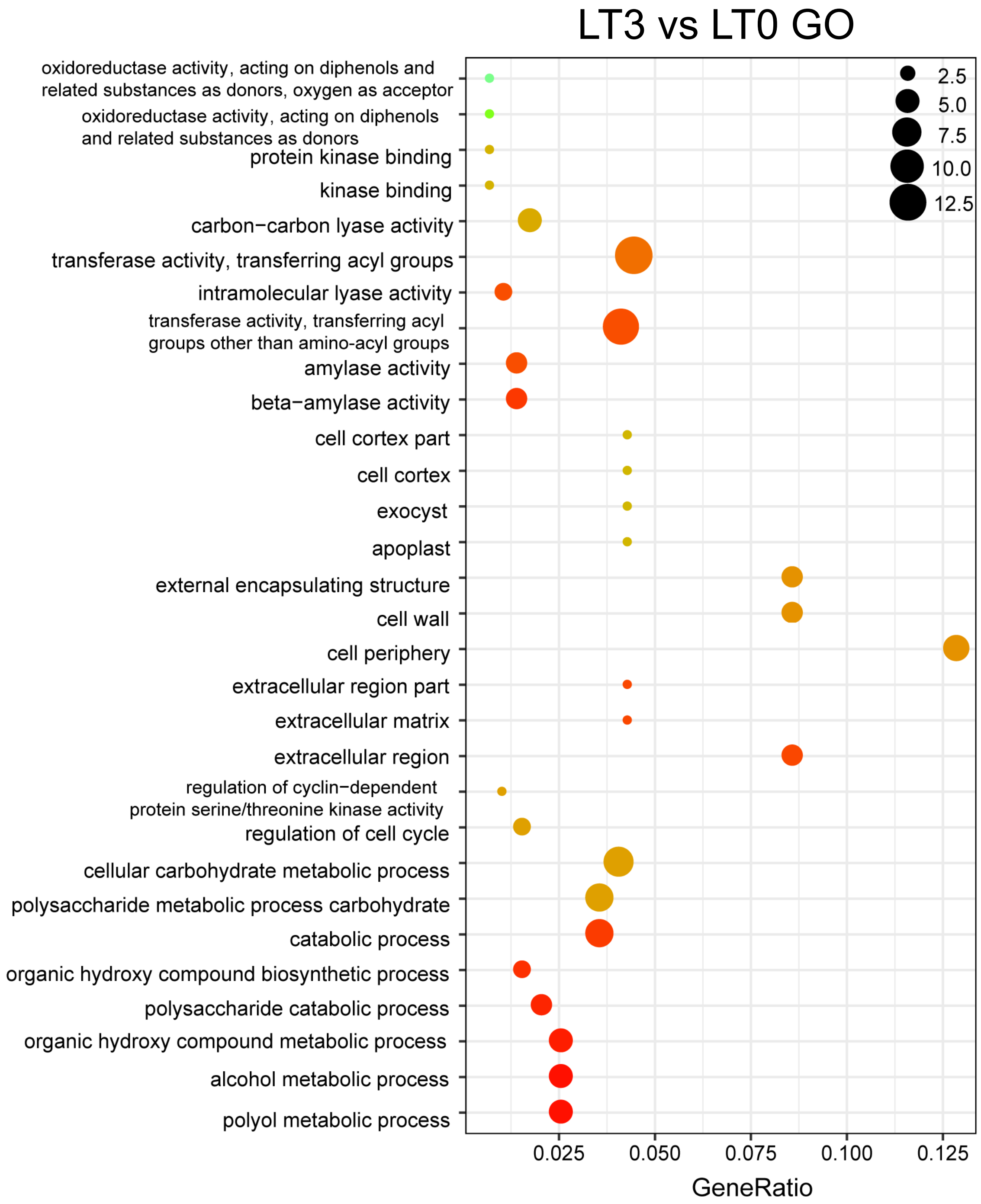
LT3 vs LT0 GO
oxidoreductase activity, acting on diphenols and related substances as donors, oxygen as acceptor
oxidoreductase activity, acting on diphenols and related substances as donors
protein kinase binding
kinase binding
carbon−carbon lyase activity
transferase activity, transferring acyl groups
intramolecular lyase activity
transferase activity, transferring acyl groups other than amino-acyl groups
amylase activity
beta−amylase activity
cell cortex part
cell cortex
exocyst
apoplast
external encapsulating structure
cell wall
cell periphery
extracellular region part
extracellular matrix
extracellular region
regulation of cyclin−dependent protein serine/threonine kinase activity
regulation of cell cycle
cellular carbohydrate metabolic process
polysaccharide metabolic process carbohydrate
catabolic process
organic hydroxy compound biosynthetic process
polysaccharide catabolic process
organic hydroxy compound metabolic process
alcohol metabolic process
polyol metabolic process
2.5
5.0
7.5
10.0
12.5
0.025
0.050
0.075
0.100
0.125
GeneRatio

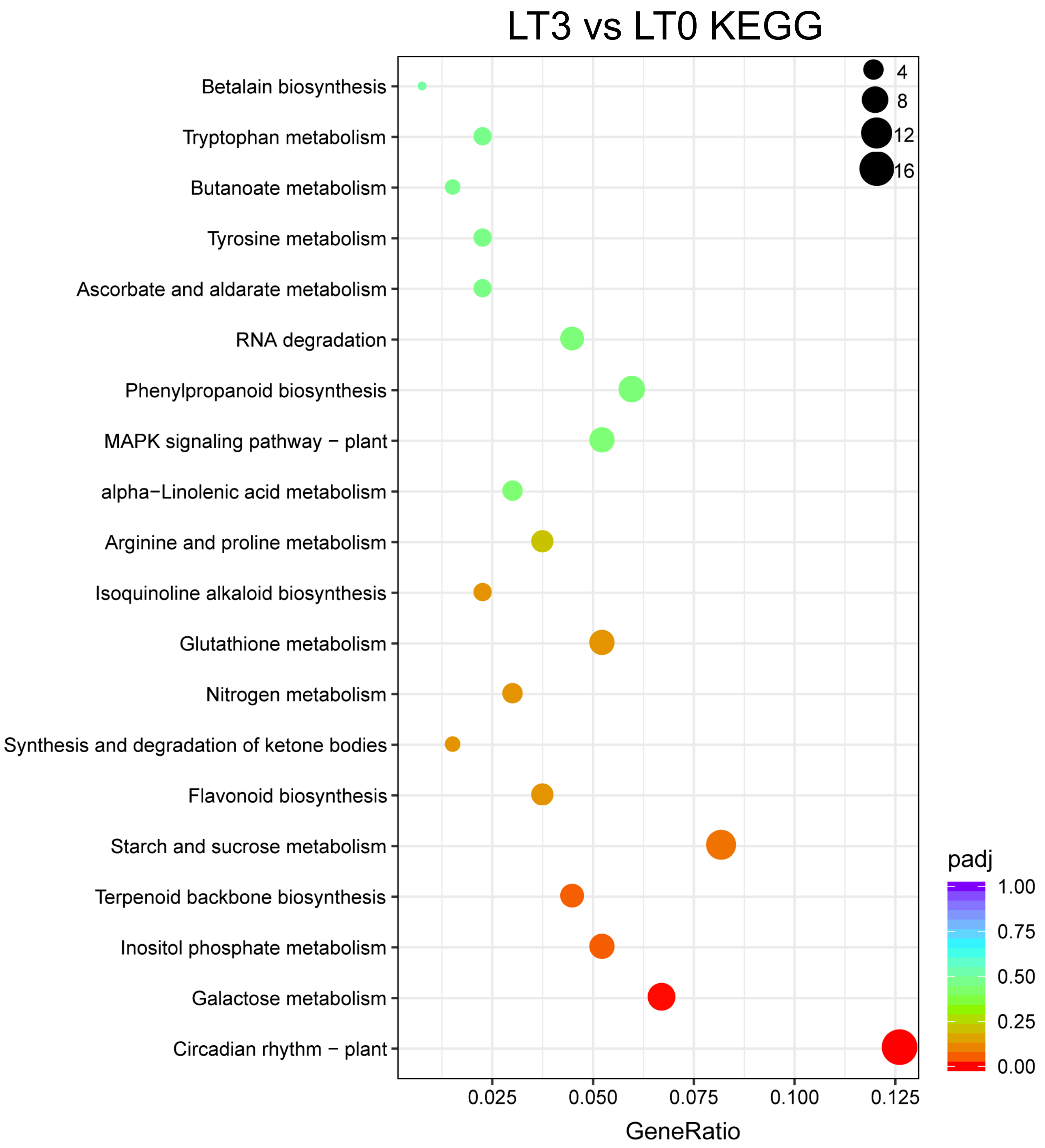
LT3 vs LT0 KEGG
Betalain biosynthesis
Tryptophan metabolism
Butanoate metabolism
Tyrosine metabolism
Ascorbate and aldarate metabolism
RNA degradation
Phenylpropanoid biosynthesis
MAPK signaling pathway − plant
alpha−Linolenic acid metabolism
Arginine and proline metabolism
Isoquinoline alkaloid biosynthesis
Glutathione metabolism
Nitrogen metabolism
Synthesis and degradation of ketone bodies
Flavonoid biosynthesis
Starch and sucrose metabolism
Terpenoid backbone biosynthesis
Inositol phosphate metabolism
Galactose metabolism
Circadian rhythm − plant
4
8
12
16
padj
1.00
0.75
0.50
0.25
0.00
0.025
0.050
0.075
0.100
0.125
GeneRatio

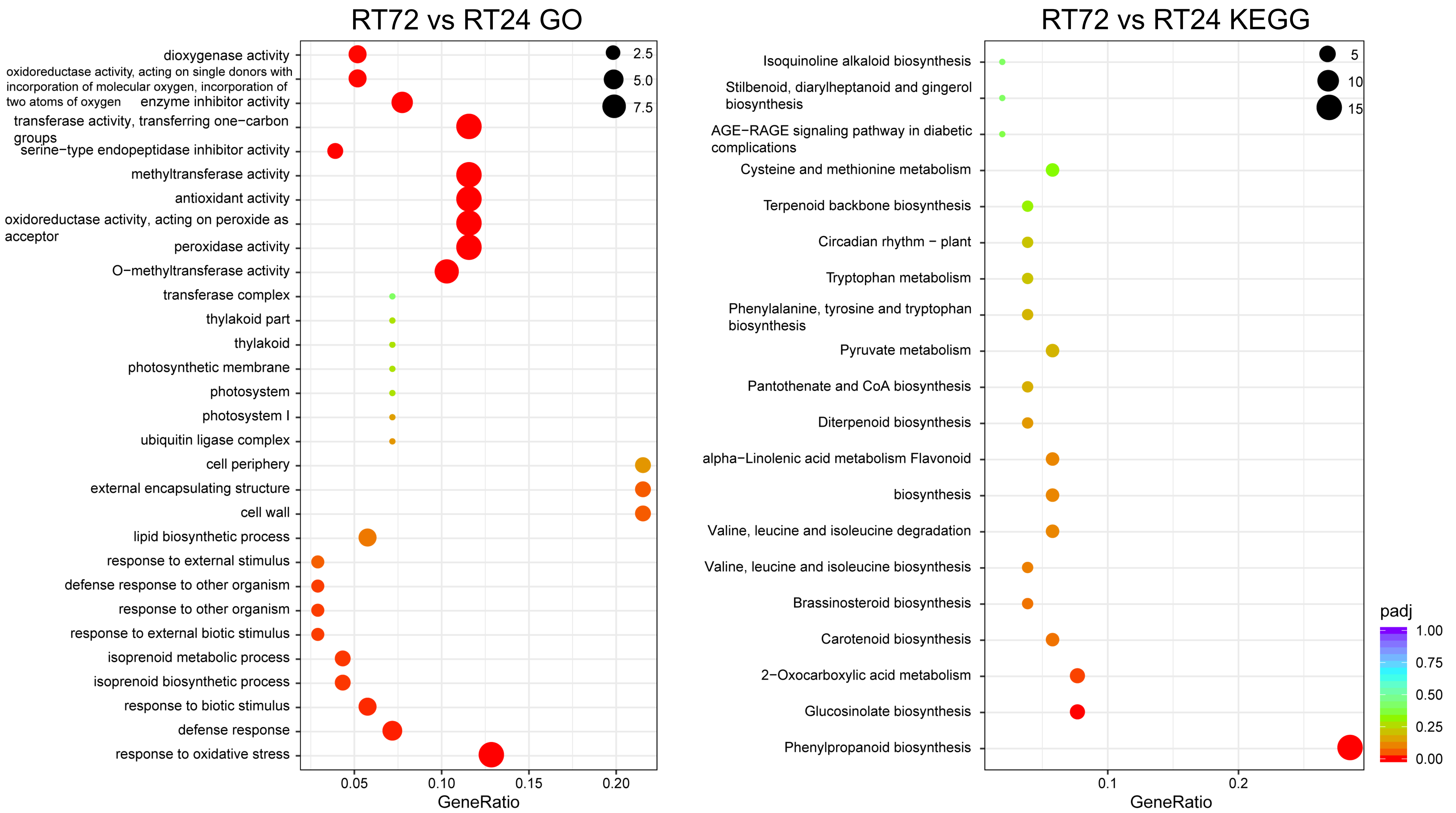
RT72 vs RT24 GO
dioxygenase activity
oxidoreductase activity, acting on single donors with incorporation of molecular oxygen, incorporation of two atoms of oxygen
enzyme inhibitor activity
transferase activity, transferring one−carbon groups
serine−type endopeptidase inhibitor activity
methyltransferase activity
antioxidant activity
oxidoreductase activity, acting on peroxide as acceptor
peroxidase activity
O−methyltransferase activity
transferase complex
thylakoid part
thylakoid
photosynthetic membrane
photosystem
photosystem I
ubiquitin ligase complex
cell periphery
external encapsulating structure
cell wall
lipid biosynthetic process
response to external stimulus
defense response to other organism
response to other organism
response to external biotic stimulus
isoprenoid metabolic process
isoprenoid biosynthetic process
response to biotic stimulus
defense response
response to oxidative stress
0.05
0.10
0.15
0.20
GeneRatio
2.5
5.0
7.5
RT72 vs RT24 KEGG
Isoquinoline alkaloid biosynthesis
Stilbenoid, diarylheptanoid and gingerol biosynthesis
AGE−RAGE signaling pathway in diabetic complications
Cysteine and methionine metabolism
Terpenoid backbone biosynthesis
Circadian rhythm − plant
Tryptophan metabolism
Phenylalanine, tyrosine and tryptophan biosynthesis
Pyruvate metabolism
Pantothenate and CoA biosynthesis
Diterpenoid biosynthesis
alpha−Linolenic acid metabolism Flavonoid
biosynthesis
Valine, leucine and isoleucine degradation
Valine, leucine and isoleucine biosynthesis
Brassinosteroid biosynthesis
Carotenoid biosynthesis
2−Oxocarboxylic acid metabolism
Glucosinolate biosynthesis
Phenylpropanoid biosynthesis
0.1
0.2
GeneRatio
5
10
15
padj
1.00
0.75
0.50
0.25
0.00

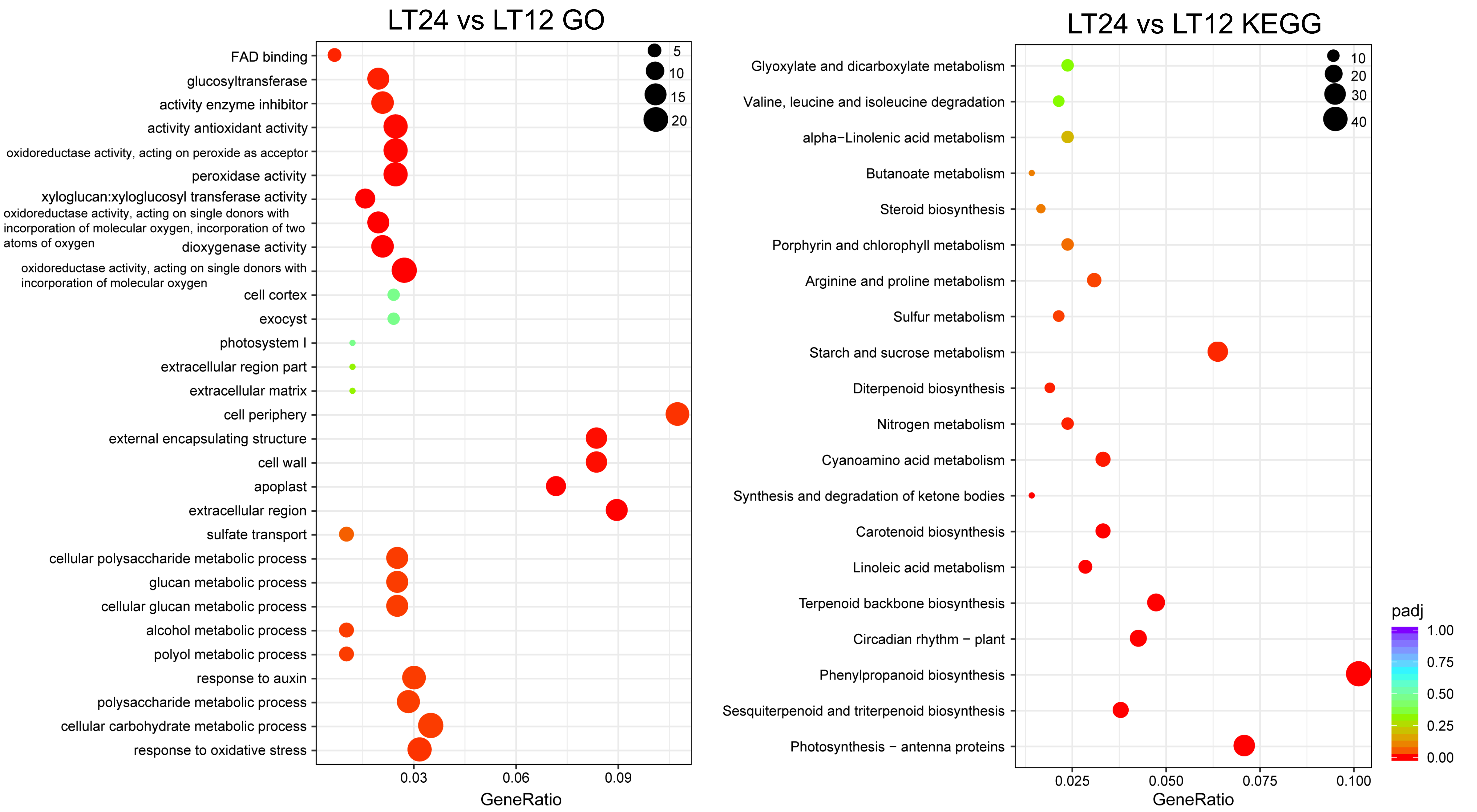
LT24 vs LT12 GO
FAD binding
glucosyltransferase
activity enzyme inhibitor
activity antioxidant activity
oxidoreductase activity, acting on peroxide as acceptor
peroxidase activity
xyloglucan:xyloglucosyl transferase activity
oxidoreductase activity, acting on single donors with incorporation of molecular oxygen, incorporation of two atoms of oxygen
dioxygenase activity
oxidoreductase activity, acting on single donors with incorporation of molecular oxygen
cell cortex
exocyst
photosystem I
extracellular region part
extracellular matrix
cell periphery
external encapsulating structure
cell wall
apoplast
extracellular region
sulfate transport
cellular polysaccharide metabolic process
glucan metabolic process
cellular glucan metabolic process
alcohol metabolic process
polyol metabolic process
response to auxin
polysaccharide metabolic process
cellular carbohydrate metabolic process
response to oxidative stress
5
10
15
20
0.03
0.06
0.09
GeneRatio
LT24 vs LT12 KEGG
Glyoxylate and dicarboxylate metabolism
Valine, leucine and isoleucine degradation
alpha−Linolenic acid metabolism
Butanoate metabolism
Steroid biosynthesis
Porphyrin and chlorophyll metabolism
Arginine and proline metabolism
Sulfur metabolism
Starch and sucrose metabolism
Diterpenoid biosynthesis
Nitrogen metabolism
Cyanoamino acid metabolism
Synthesis and degradation of ketone bodies
Carotenoid biosynthesis
Linoleic acid metabolism
Terpenoid backbone biosynthesis
Circadian rhythm − plant
Phenylpropanoid biosynthesis
Sesquiterpenoid and triterpenoid biosynthesis
Photosynthesis − antenna proteins
10
20
30
40
padj
1.00
0.75
0.50
0.25
0.00
0.025
0.050
0.075
0.100
GeneRatio

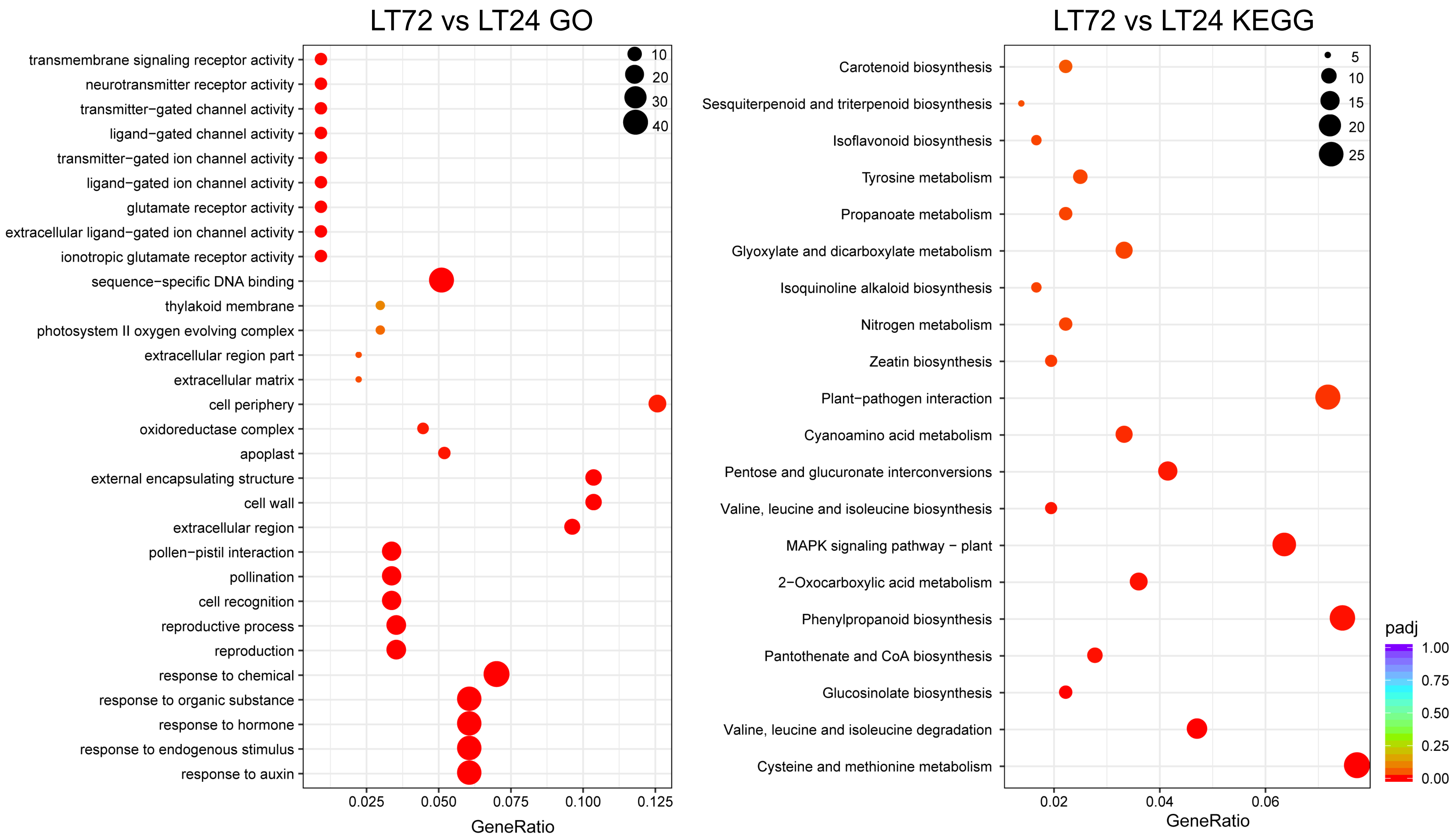

LT72 vs LT24 GO
transmembrane signaling receptor activity
neurotransmitter receptor activity
transmitter−gated channel activity
ligand−gated channel activity
transmitter−gated ion channel activity
ligand−gated ion channel activity
glutamate receptor activity
extracellular ligand−gated ion channel activity
ionotropic glutamate receptor activity
sequence−specific DNA binding
thylakoid membrane
photosystem II oxygen evolving complex
extracellular region part
extracellular matrix
cell periphery
oxidoreductase complex
apoplast
external encapsulating structure
cell wall
extracellular region
pollen−pistil interaction
pollination
cell recognition
reproductive process
reproduction
response to chemical
response to organic substance
response to hormone
response to endogenous stimulus
response to auxin
10
20
30
40
0.025
0.050
0.075
0.100
0.125
GeneRatio
LT72 vs LT24 KEGG
Carotenoid biosynthesis
Sesquiterpenoid and triterpenoid biosynthesis
Isoflavonoid biosynthesis
Tyrosine metabolism
Propanoate metabolism
Glyoxylate and dicarboxylate metabolism
Isoquinoline alkaloid biosynthesis
Nitrogen metabolism
Zeatin biosynthesis
Plant−pathogen interaction
Cyanoamino acid metabolism
Pentose and glucuronate interconversions
Valine, leucine and isoleucine biosynthesis
MAPK signaling pathway − plant
2−Oxocarboxylic acid metabolism
Phenylpropanoid biosynthesis
Pantothenate and CoA biosynthesis
Glucosinolate biosynthesis
Valine, leucine and isoleucine degradation
Cysteine and methionine metabolism
5
10
15
20
25
0.02
0.04
0.06
GeneRatio
padj
1.00
0.75
0.50
0.25
0.00

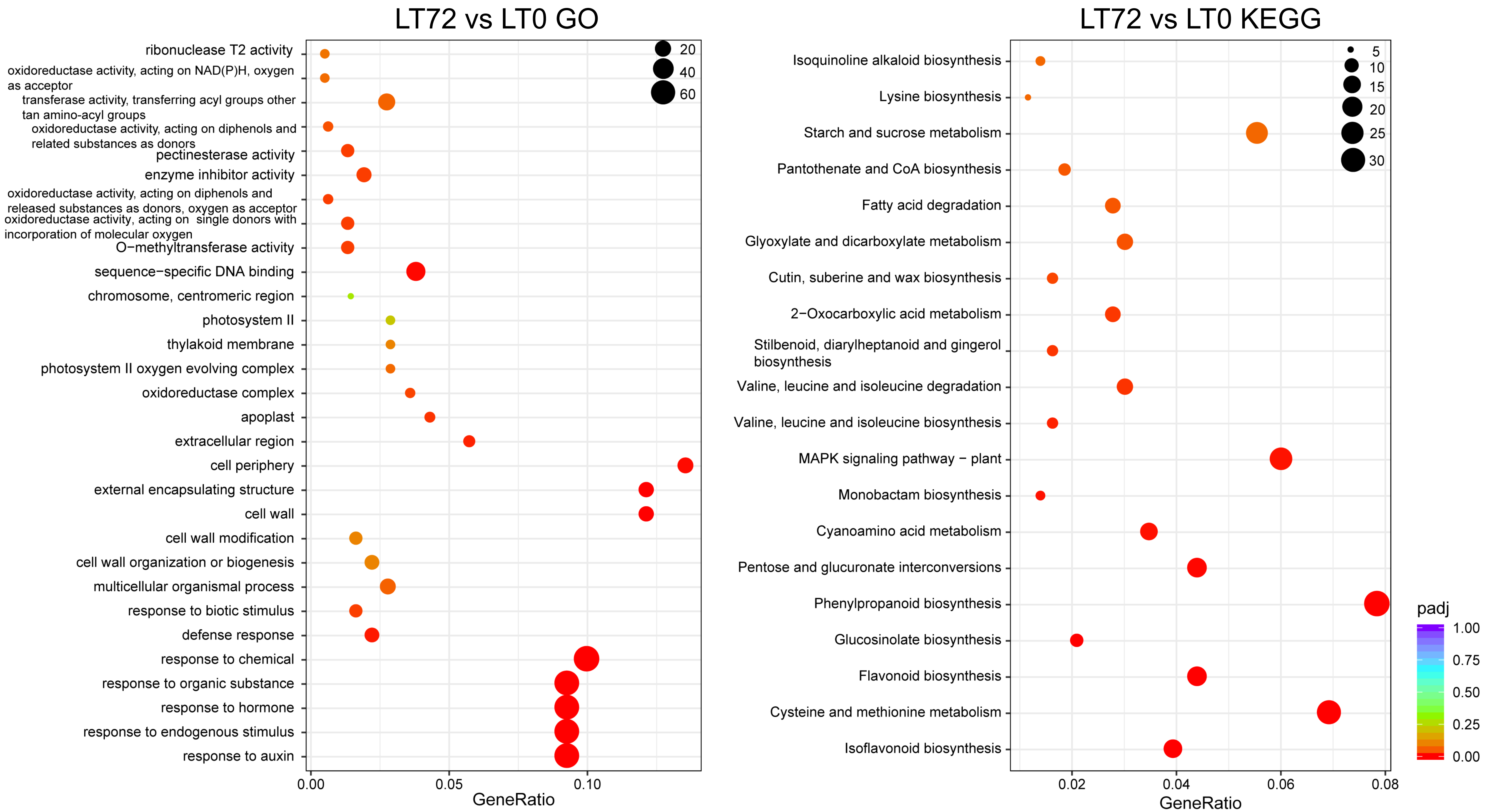

LT72 vs LT0 GO
ribonuclease T2 activity
oxidoreductase activity, acting on NAD(P)H, oxygen as acceptor
transferase activity, transferring acyl groups other tan amino-acyl groups
oxidoreductase activity, acting on diphenols and related substances as donors
pectinesterase activity
enzyme inhibitor activity
oxidoreductase activity, acting on diphenols and released substances as donors, oxygen as acceptor
oxidoreductase activity, acting on single donors with incorporation of molecular oxygen
O−methyltransferase activity
sequence−specific DNA binding
chromosome, centromeric region
photosystem II
thylakoid membrane
photosystem II oxygen evolving complex
oxidoreductase complex
apoplast
extracellular region
cell periphery
external encapsulating structure
cell wall
cell wall modification
cell wall organization or biogenesis
multicellular organismal process
response to biotic stimulus
defense response
response to chemical
response to organic substance
response to hormone
response to endogenous stimulus
response to auxin
20
40
60
0.00
0.05
0.10
GeneRatio
LT72 vs LT0 KEGG
Isoquinoline alkaloid biosynthesis
Lysine biosynthesis
Starch and sucrose metabolism
Pantothenate and CoA biosynthesis
Fatty acid degradation
Glyoxylate and dicarboxylate metabolism
Cutin, suberine and wax biosynthesis
2−Oxocarboxylic acid metabolism
Stilbenoid, diarylheptanoid and gingerol biosynthesis
Valine, leucine and isoleucine degradation
Valine, leucine and isoleucine biosynthesis
MAPK signaling pathway − plant
Monobactam biosynthesis
Cyanoamino acid metabolism
Pentose and glucuronate interconversions
Phenylpropanoid biosynthesis
Glucosinolate biosynthesis
Flavonoid biosynthesis
Cysteine and methionine metabolism
Isoflavonoid biosynthesis
5
10
15
20
25
30
padj
1.00
0.75
0.50
0.25
0.00
0.02
0.04
0.06
0.08
GeneRatio
