# Supplementary figures and images for: Unraveling Cadmium Toxicity in Trifolium repens L. Seedling: Insight into Regulatory Mechanisms Using Comparative Transcriptomics Combined with Physiological Analyses
Source: Int J Mol Sci. 2022 Apr 21;23(9):4612. doi: 10.3390/ijms23094612 (PMC9105629; doi:10.3390/ijms23094612)

**(a)**

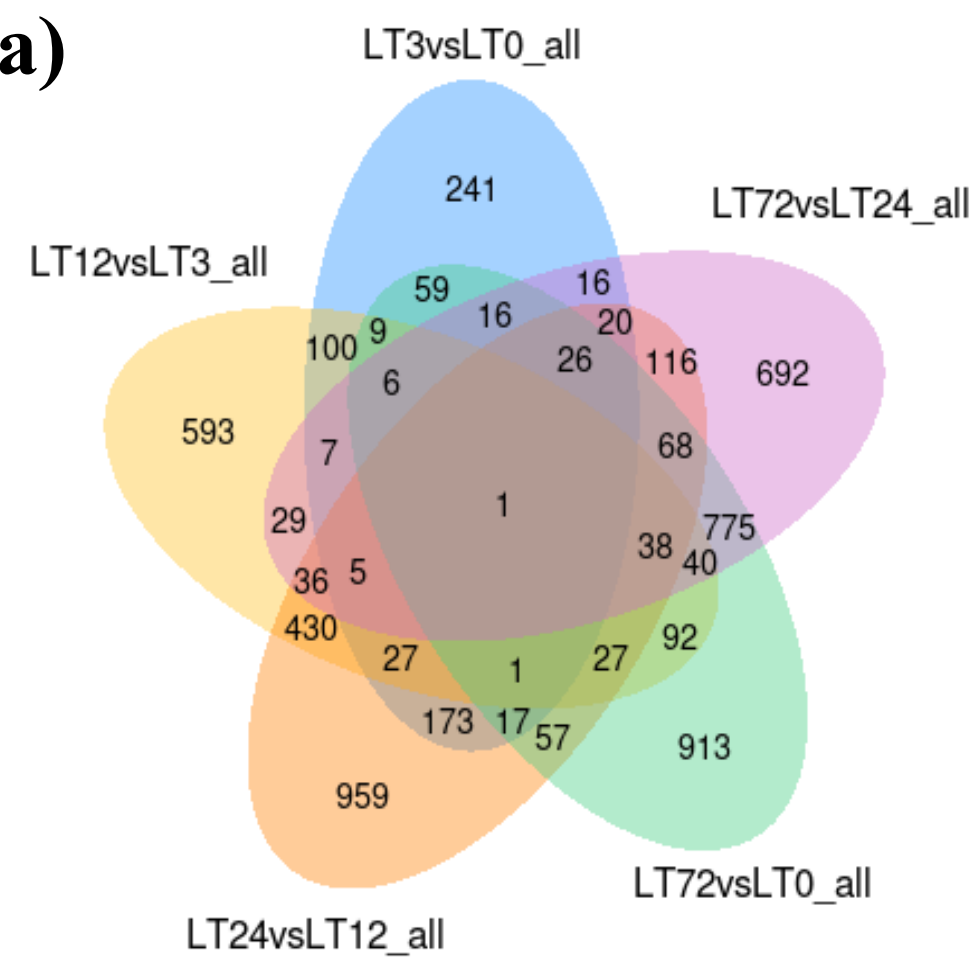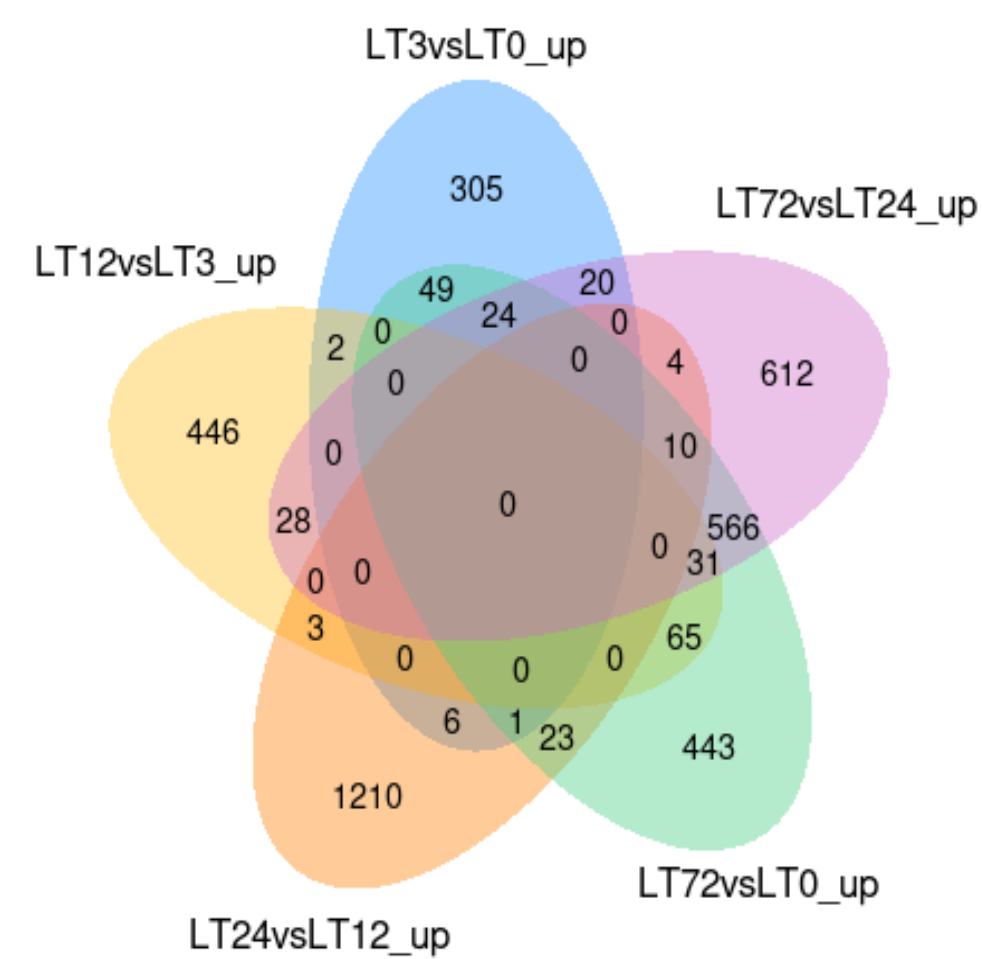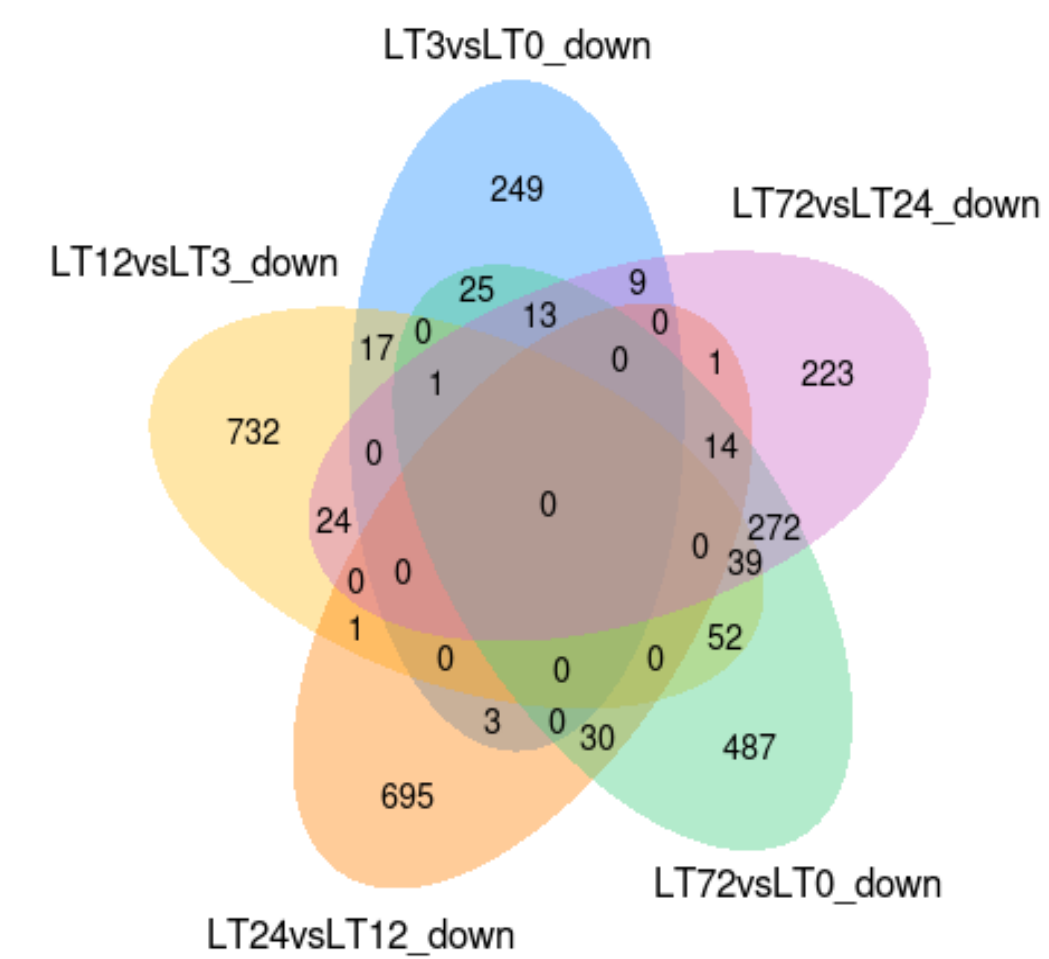

**(b)**

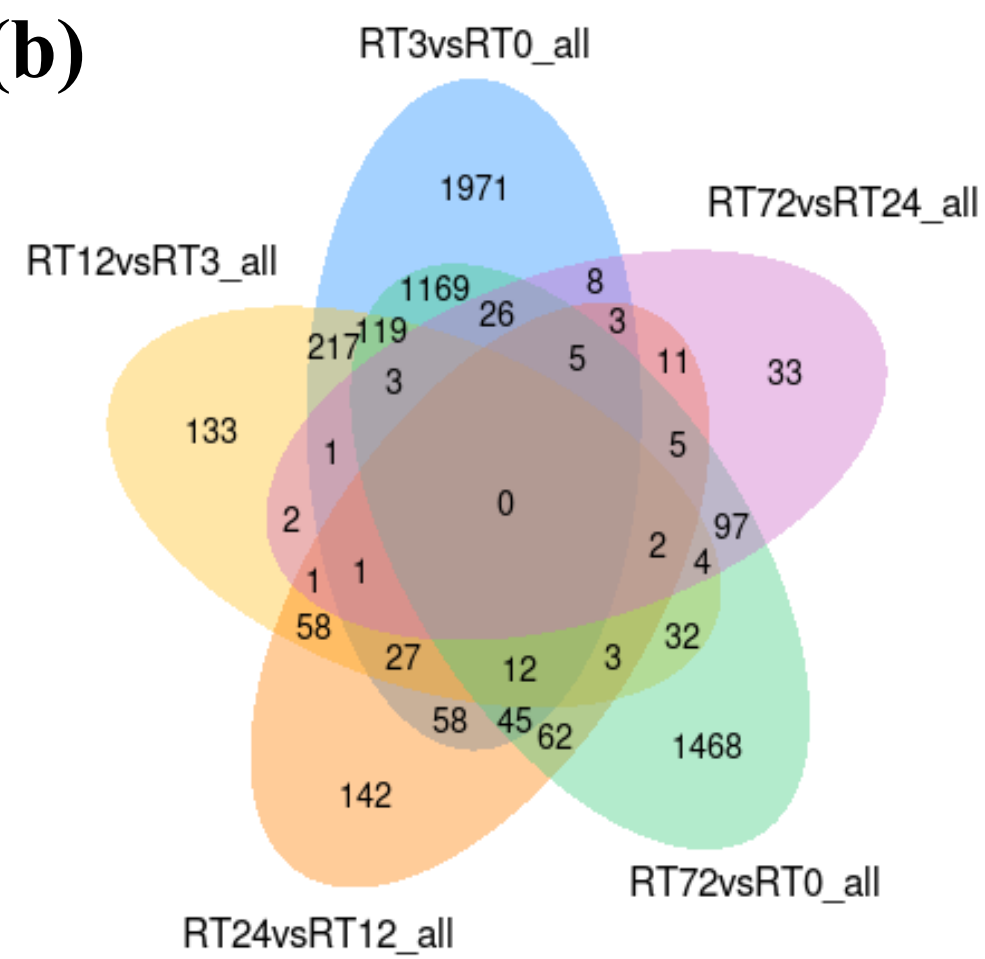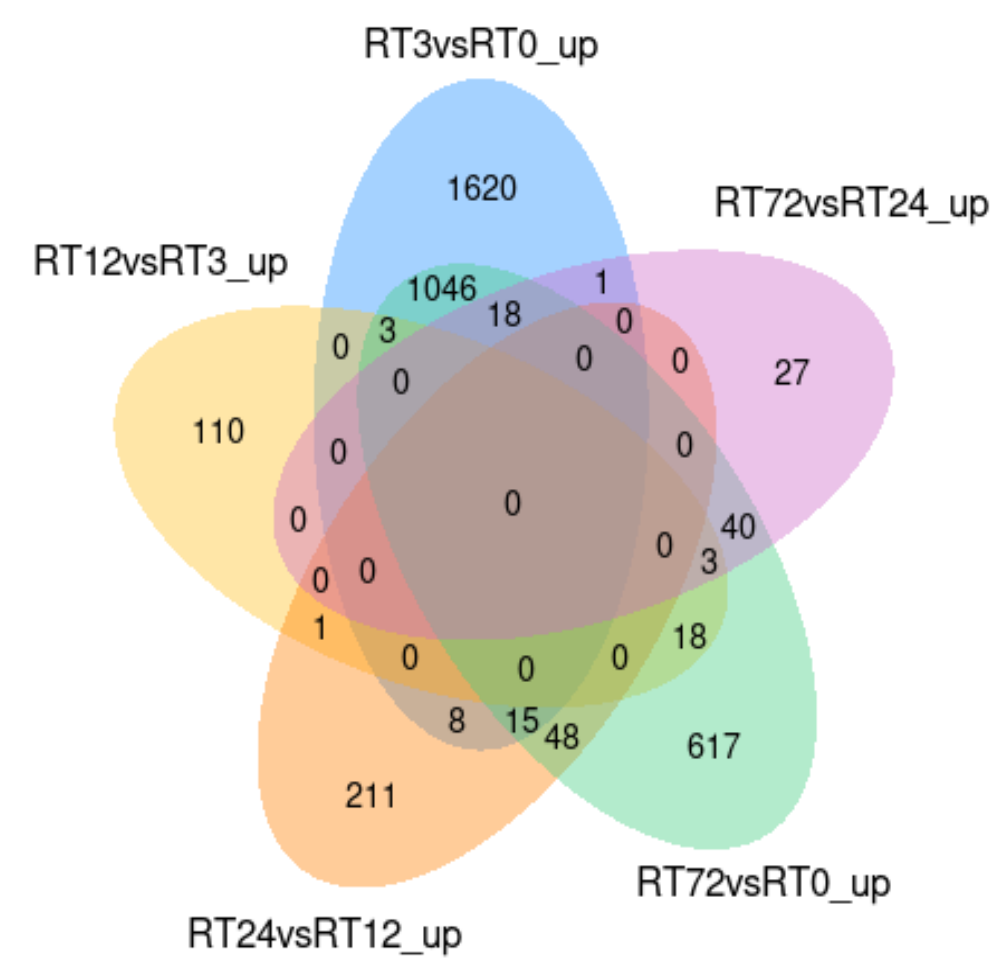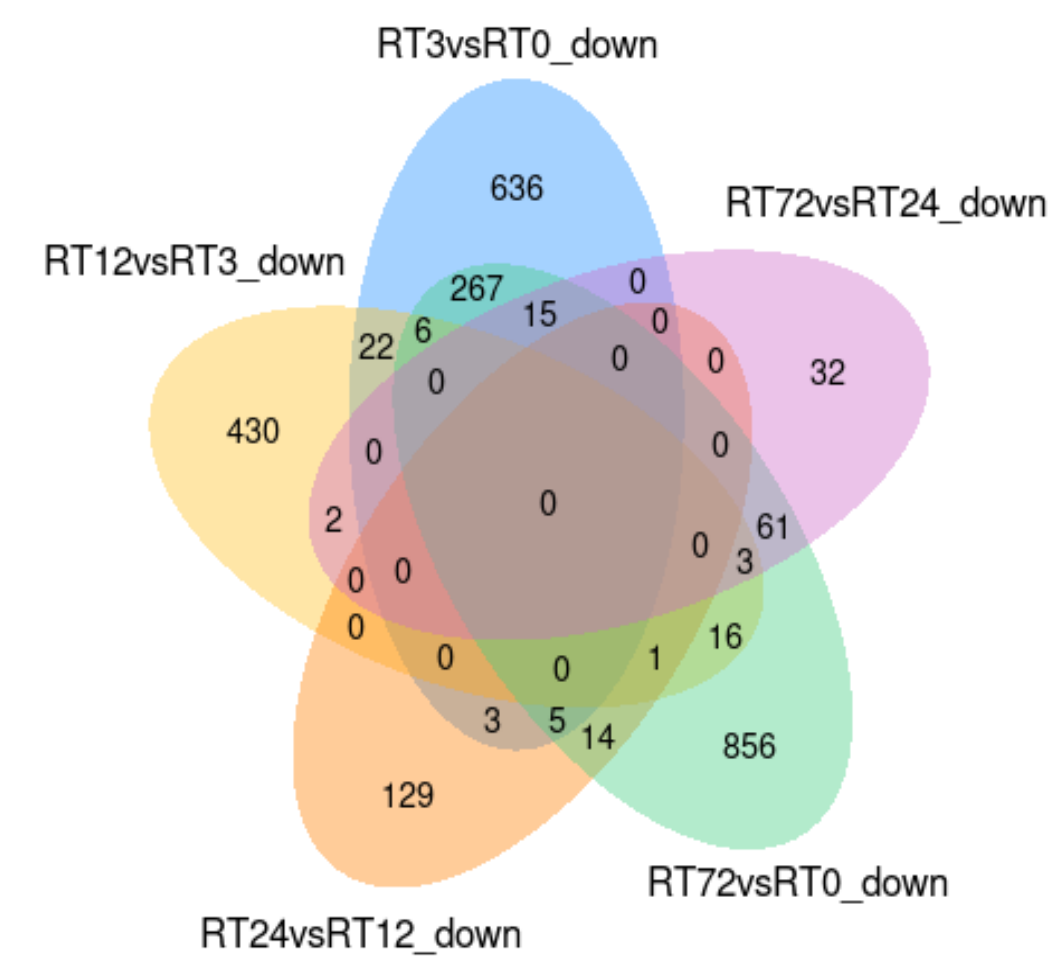

**(c)**

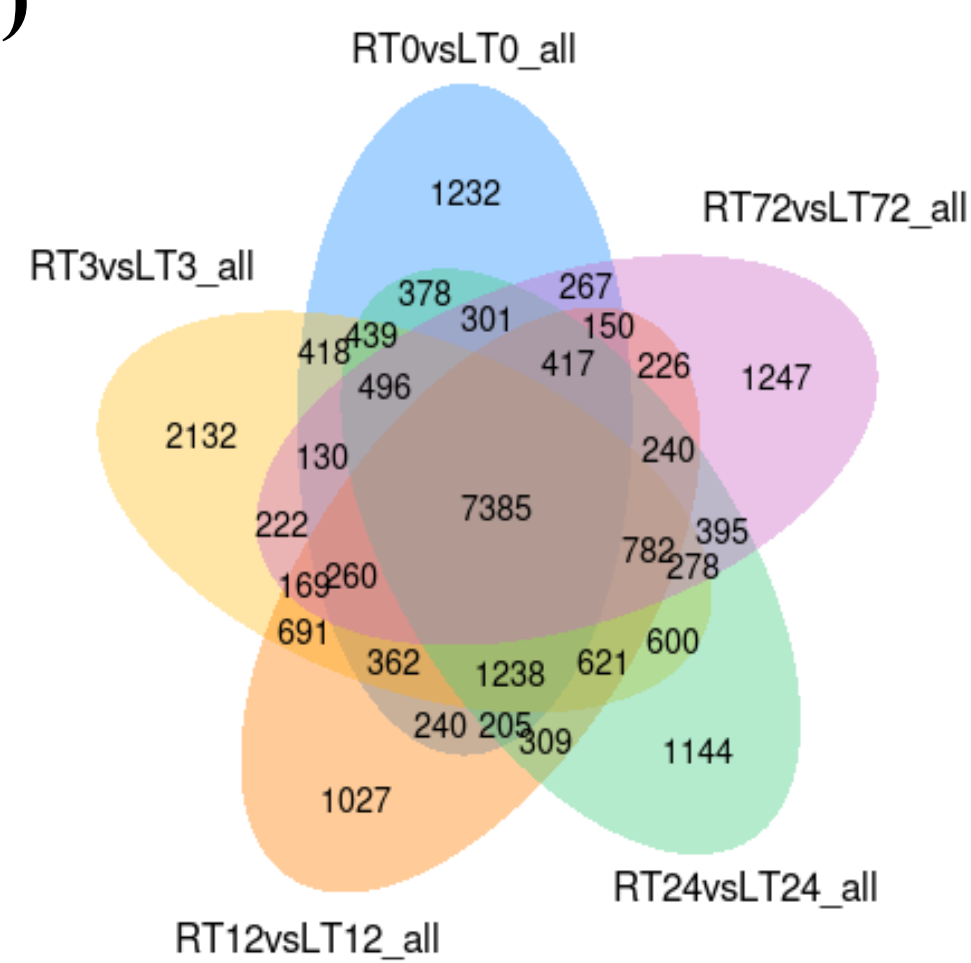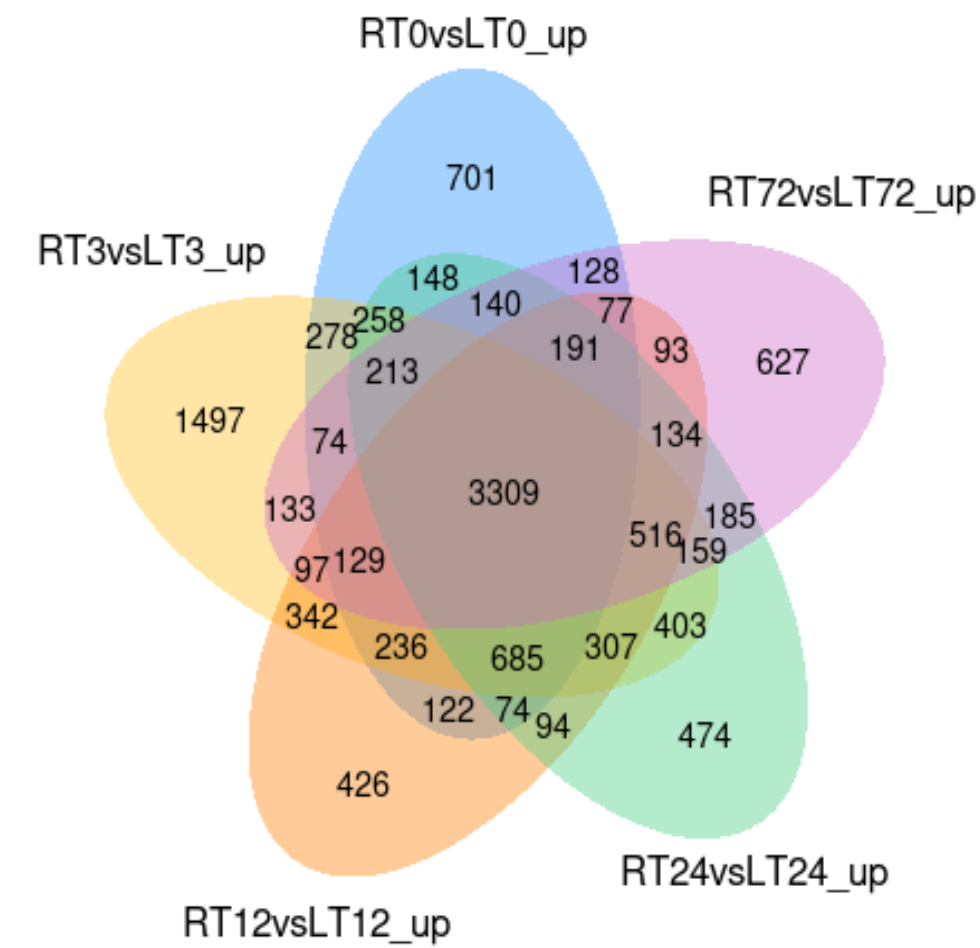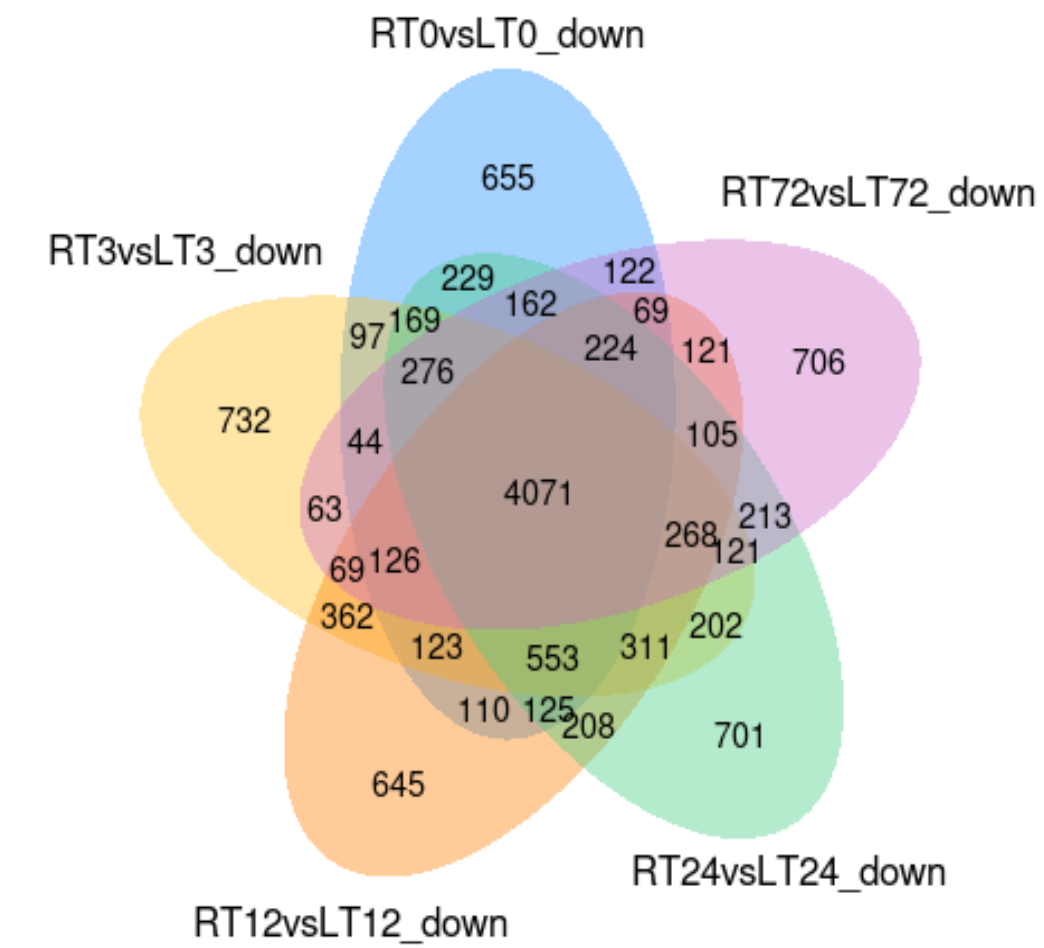

Supplement: Supplementary file 1 [file ijms-23-04612-s001.zip › ijms-1682701-supplementary/Figure S1. Venn.pdf]
